# Gene Duplication and Differential Expression of Flower Symmetry Genes in *Rhododendron* (Ericaceae)

**DOI:** 10.3390/plants10101994

**Published:** 2021-09-23

**Authors:** Elizabeth Ramage, Valerie L. Soza, Jing Yi, Haley Deal, Vaidehi Chudgar, Benjamin D. Hall, Verónica S. Di Stilio

**Affiliations:** 1 Department of Biology, University of Washington, Seattle, WA 98195, USA; bethramage@gmail.com (E.R.); h.deal23@gmail.com (H.D.); vaidehic@uw.edu (V.C.); benhall@uw.edu (B.D.H.); distilio@uw.edu (V.S.D.S.); 2 Key Laboratory of Ecology and Environmental Science in Guangdong Higher Education, School of Life Science, South China Normal University, Guangzhou 510631, China; jingyi@umass.edu

**Keywords:** *CYCLOIDEA*, *DIVARICATA*, *RADIALIS*, paralogs, sect. *Schistanthe*, vireya, tandem duplication

## Abstract

Bilaterally symmetric flowers have evolved over a hundred times in angiosperms, yet orthologs of the transcription factors *CYCLOIDEA* (*CYC*), *RADIALIS* (*RAD*), and *DIVARICATA* (*DIV*) are repeatedly implicated in floral symmetry changes. We examined these candidate genes to elucidate the genetic underpinnings of floral symmetry changes in florally diverse *Rhododendron*, reconstructing gene trees and comparing gene expression across floral organs in representative species with radial and bilateral flower symmetries. Radially symmetric *R. taxifolium* Merr. and bilaterally symmetric *R. beyerinckianum* Koord. had four and five *CYC* orthologs, respectively, from shared tandem duplications. *CYC* orthologs were expressed in the longer dorsal petals and stamens and highly expressed in *R. beyerinckianum* pistils, whereas they were either ubiquitously expressed, lost from the genome, or weakly expressed in *R. taxifolium*. Both species had two *RAD* and *DIV* orthologs uniformly expressed across all floral organs. Differences in gene structure and expression of *Rhododendron RAD* compared to other asterids suggest that these genes may not be regulated by *CYC* orthologs. Our evidence supports *CYC* orthologs as the primary regulators of differential organ growth in *Rhododendron* flowers, while also suggesting certain deviations from the typical asterid gene regulatory network for flower symmetry.

## 1. Introduction

Flower symmetry is a feature of angiosperms that influences pollinator attraction, visitation, and overall specialization [1]. In contrast to radial symmetry, which exhibits multiple planes of symmetry, bilateral flower symmetry has a single dorsoventral plane of symmetry and is associated with increased species richness [2] and increased diversification rates [3]. Fossil records suggest that bilateral symmetry evolved in various lineages at least 50 my after the origin of angiosperms, when specialized insect pollinators were diversifying [4]. Bilateral symmetry has evolved at least 130 times from radial symmetry in angiosperms [5], potentially due to pollinator-mediated selection.

The gene regulatory network controlling floral symmetry in the asterid *Antirrhinum majus* L. (snapdragon) is a model for bilateral symmetry development in other angiosperms. The best characterized candidate genes for floral symmetry are transcription factors belonging to the TEOSINTE BRANCHED1/CYCLOIDEA/PROLIFERATING CELL FACTOR (TCP) gene family, *CYCLOIDEA* (*CYC*). and its paralog *DICHOTOMA* (*DICH*) [6,7,8,9,10,11], and the MYELOBLASTOSIS (MYB) family, *RADIALIS (RAD),* and *DIVARICATA (DIV)* [12,13]. *CYC* and *DICH* are dorsal identity genes initially expressed dorsally within the floral meristem and later expressed in dorsal petals and staminode [6,8]. *DICH* expression is further restricted to the dorsal half of each dorsal petal [8]. CYC and DICH activate the transcription of their direct target *RAD* by binding to its promoter and intron [14]. *RAD* is also expressed in dorsal petals and staminodes [13,15] and acts cell-non-autonomously in lateral petals to suppress ventralizing DIV function in both dorsal and lateral petals [13].

The TCP gene family encodes plant-specific DNA-binding transcription factors that are involved in cell growth and division [7,16]. TCP genes in angiosperms that contain a TCP domain [7], an arginine-rich (R) domain [7], and a glutamic acid–cysteine–glutamic acid (ECE) motif [17] are classified as the ECE clade [18]. Within the ECE clade, three paralogous clades are found in the core eudicots: CYC1–3 [18]. In core eudicots, floral symmetry changes are associated with genes from the CYC2 lineage [18], which have a conserved LxxLL amino acid motif in the second helix of the TCP domain [19]. Genes in this clade have undergone independent duplications, as in *Antirrhinum* (*CYC*/*DICH*), and have been repeatedly recruited for floral symmetry function [4,20]. In bilaterally symmetric core eudicots, similar patterns have emerged where one paralog from the CYC2 lineage is more dorsally restricted than the other paralog(s), suggesting that independent evolution of bilateral symmetry has occurred through repeated restrictions of *CYC* ortholog expression [21]. Additionally, genes from the CYC2 lineage have independently shifted between dorsally restricted expression in bilaterally symmetric flowers to loss of expression or ubiquitous expression in radially symmetric flowers [4,21,22,23].

MYB proteins typically contain one to three MYB domains [24], with DIV containing two MYB domains (MYBI-II) and RAD containing one [25]. Three paralogous clades of *RAD*-like (RAD1–3) and *DIV*-like (DIV1–3/EudiDIV1–3) genes have also been identified in eudicots [26,27,28], although genes from the RAD1 and RAD3 lineages were not respectively monophyletic in recent studies [28,29]. Genes from the RAD2 and DIV1 lineages are involved in floral symmetry control in *Antirrhinum*, as described above [26,27]. Dorsal expression of *RAD* orthologs is shared across bilaterally symmetric asterid flowers [27,30,31,32]. Additionally, duplications in *RAD* orthologs have been associated with duplications in *CYC* orthologs [27]. Similarly, loss of expression or function of *CYC* orthologs in radially symmetric asterid flowers is correlated with loss of expression or function of *RAD* orthologs [30,32,33,34,35,36]. In other cases, ubiquitous expression of *RAD* orthologs, in conjunction with a *CYC* ortholog, is correlated with radial symmetry [32,37]. In rosids, however, *RAD* orthologs are not expressed dorsally in flowers; instead, they are expressed in other reproductive and/or vegetative tissue [38,39,40,41]. Therefore, the co-option of *RAD* orthologs to floral symmetry may be asterid specific [4].

In *Antirrhinum, DIV* is expressed in all floral organs, but its activity is reduced in dorsal regions later in flower development, likely due to post-transcriptional inhibition [12]. *DIV* has duplicated in *Antirrhinum* to produce *DIV-LIKE1* (*DVL1*) [26,28], which is also expressed in all floral organs at early stages but becomes expressed mainly in ovules later in development [12]. Similarly, in other asterids, *DIV* orthologs have duplicated and diverged in their expression patterns, with one paralog expressed throughout all floral organs and the other in dorsal and ventral petals [26]. Duplications in *CYC* orthologs likely have cascading effects that result in duplications of their interacting *RAD* and *DIV* orthologs [17,26,27], warranting their joined investigation in more asterids.

**Figure 1:**
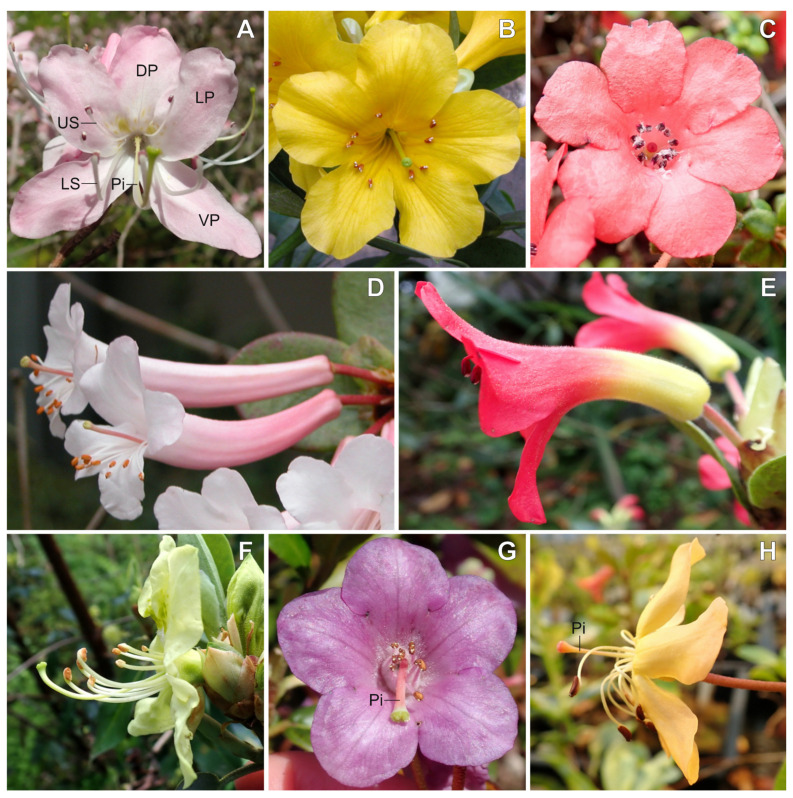
Flower symmetry variation across *Rhododendron* (Ericaceae). (**A**) Bilateral corolla symmetry and abaxially curved stamens and style in *R. vaseyi* A. Gray, with five petals, as is typical for the genus. (**B**) Radial corolla symmetry in *R. laetum* J.J.Sm. (**C**) Radial corolla symmetry due to merosity change (six petals) and dimorphic stamen length in *R. rubineiflorum* Craven. (**D**) Abaxially curved corolla tube in *R. tuba*. (**E**) Adaxially curved corolla tube in *R. christi* F.Först. (**F**) Lower stamens longer than upper stamens and abaxially curved stamens and style in *R. triflorum* Hook. f. var. *bauhiniiflorum* (Watt ex Hutch.) Cullen in Cullen and D. F. Chamb. (**G**) Radial corolla symmetry and adaxially curved style in *R. charitopes* Balf. f. and Farrer subsp. *tsangpoense* (Kingdon-Ward) Cullen. (**H**) Straight style in *R. macgregoriae* F.Muell. DP = dorsal petal, LP = lateral petal, VP = ventral petal, US = upper stamen, LS = lower stamen, Pi = pistil.

The genus *Rhododendron*, which includes azaleas, contains over 1000 wild species that exhibit variation in flower symmetry [42] and belongs to the order Ericales, an early-diverging lineage of asterids [43]. Five subgenera have been identified based on molecular phylogenetic studies [44,45]. Using these data, multiple transitions between bilaterally and radially symmetric corollas have been reconstructed within the main clades [46]. However, variation in flower symmetry in this group is achieved through structural symmetry variation in stamens, styles, and petals, all of which are often curved (Figure 1). In fact, correlated evolution among floral organ symmetry has been shown in the group [46], between curvature of the corolla, stamens, and/or styles. Many temperate *Rhododendron* species exhibit stamens and styles that are abaxially positioned (Figure 1A,F), aiding in pollen deposition on the underside of the pollinator [47]. In contrast, tropical species in sect. *Schistanthe* [48], known as vireyas, exhibit a unique form of bilateral symmetry compared to temperate species. Some have evolved red, adaxially curved corolla tubes and adaxially positioned stamens (Figure 1E) that are thought to be bird-pollinated, aiding in pollen deposition on the back of the pollinator [47].

Here, we set out to investigate whether conserved patterns of gene expression of *CYC*, *RAD*, and *DIV* orthologs in asterids were also found in *Rhododendron*. Our hypothesis was that duplication and/or loss of *CYC* orthologs played a role in the evolution of flower symmetry in *Rhododendron*. We predicted that (1) *CYC* orthologs underwent gene duplication and subsequent loss in *Rhododendron*, (2) *RAD* and *DIV* orthologs subsequently underwent compensatory duplication and/or loss, and (3) paralogs evolved different expression patterns across floral organs (petals, stamens, and pistils) in radially versus bilaterally symmetric species. To that end, we isolated *CYC, RAD,* and *DIV* orthologs from eight *Rhododendron* species across four subgenera to reconstruct gene trees in order to identify gene duplications and losses across the genus. We then selected two closely related species from sect. *Schistanthe* that exhibit different floral symmetries and compared expression patterns of paralogs across different developmental stages and floral organs.

## 2. Results

### 2.1. Characterization of CYC, RAD, and DIV Orthologs in Rhododendron

**Figure 2:**
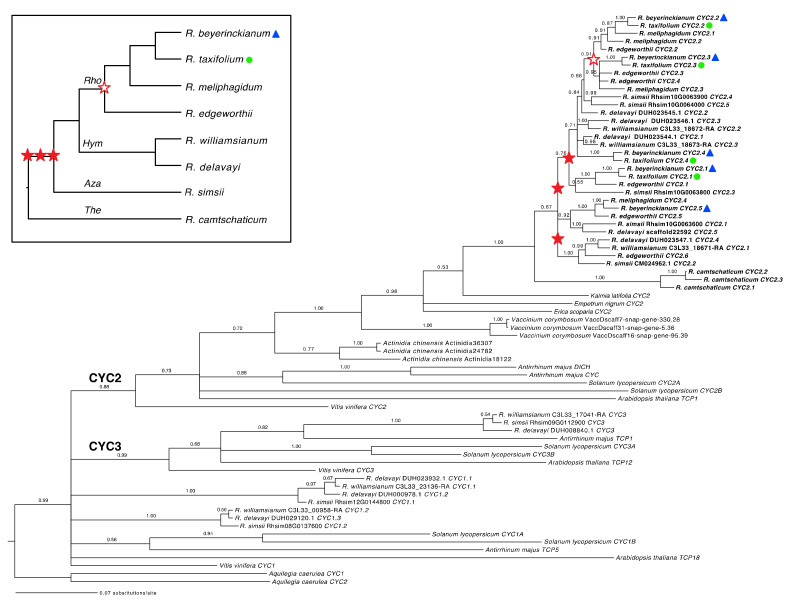
Evolution of *CYCLOIDEA* (*CYC*) homologs in *Rhododendron* (Ericaceae) and outgroups. Reconstructed Bayesian *CYC* phylogeny with posterior probabilities at all nodes. Three gene duplications in the CYC2 lineage that are shared by subgenera *Azaleastrum, Hymenanthes*, and *Rhododendron*, indicated by filled red stars, occurred in *Rhododendron* history after its divergence from *R. camtschaticum*. A subsequent lineage-specific duplication in subgenus *Rhododendron* is indicated by an empty red star. Experimental species are highlighted with a blue triangle (bilateral symmetry) and green circle (radial symmetry). Inset shows sampled *Rhododendron* taxa in phylogenetic context, based on prior studies [44,45,55]. *Aza* = subg. *Azaleastrum*, *Hym* = subg. *Hymenanthes*, *Rho* = subg. *Rhododendron*, and *The* = subg. *Therorhodion*.

**Figure 3:**
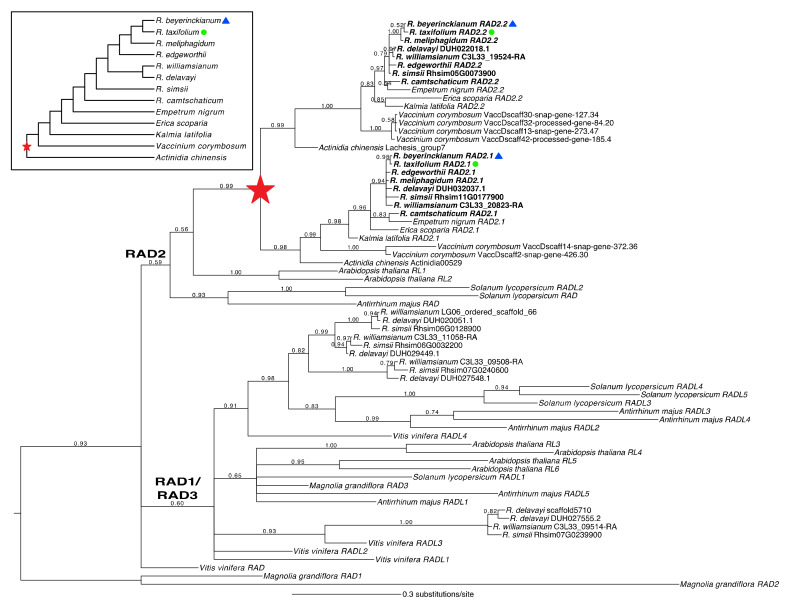
Evolution of *RADIALIS* (*RAD*) homologs in *Rhododendron* (Ericaceae) and outgroups. Reconstructed Bayesian *RAD* phylogeny with posterior probabilities at all nodes. One gene duplication in the RAD2 lineage, indicated by a filled red star, occurred in an ancestor of the Ericales. Experimental species are highlighted with a blue triangle (bilateral symmetry) and a green circle (radial symmetry). Inset shows sampled taxa in phylogenetic context, based on prior studies [44,45,55,56,57].

**Figure 4:**
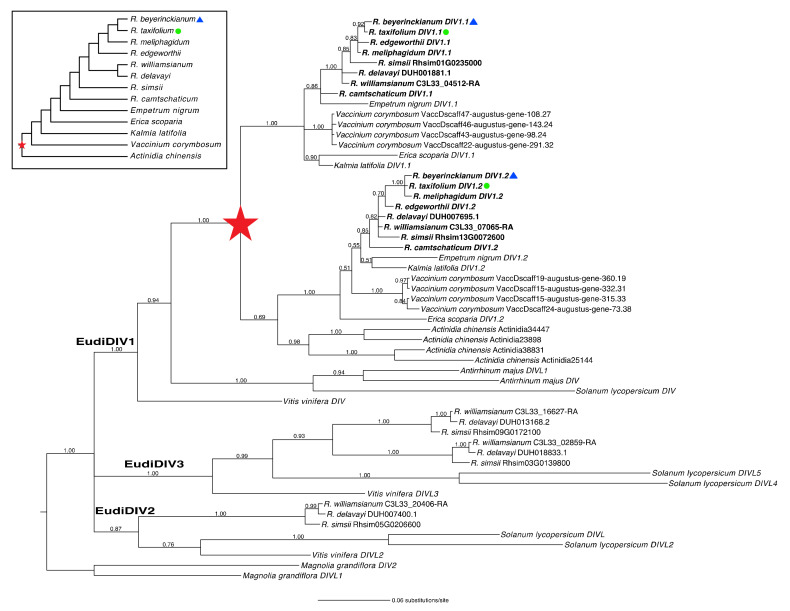
Evolution of *DIVARICATA* (*DIV*) homologs in *Rhododendron* (Ericaceae) and outgroups. Reconstructed Bayesian *DIV* phylogeny with posterior probabilities at all nodes. One gene duplication in the EudiDIV1 (DIV1) lineage, indicated by a filled red star, occurred in an ancestor of the Ericales. Experimental species are highlighted with a blue triangle (bilateral symmetry) and a green circle (radial symmetry). Inset shows sampled taxa in phylogenetic context, based on prior studies [44,45,55,56,57].

To obtain orthologs of the flower symmetry candidate genes *CYC*, *RAD*, and *DIV*, we used publicly available genomes [49,50,51,52,53], polymerase chain reaction (PCR), and/or in-house transcriptomes [54] from eight *Rhododendron* species across four subgenera (Figure 2, inset) and five Ericales outgroups (Figure 3 and Figure 4, inset), based on prior studies [44,45,55,56,57] (Supplementary Tables S1–S3). Sampling included two experimental species with different floral symmetries, *R. beyerinckianum* Koord. (bilateral) and *R. taxifolium* Merr. (radial). Sequences were analyzed in a phylogenetic context with other publicly available sequences (Supplementary Table S2) to verify orthology and identify gene duplications and losses.

#### 2.1.1. Both Shared and Independent Duplications and Losses in *Rhododendron CYC* Orthologs

The inclusion of sequences from the ECE clade from other asterids and rosids allowed us to identify *Rhododendron* orthologs for all three CYC lineages (CYC1–3). CYC2 and CYC3 lineages formed monophyletic groups with posterior probabilities (PPs) of 0.88 and 0.99, respectively, but the CYC1 lineage was not recovered as a clade (Figure 2). From our expanded sampling of *Rhododendron* species and Ericales outgroups in the CYC2 clade, we found lineage-specific duplications in subgenus *Therorhodion*, sister to the remaining members of the genus, resulting in three paralogs in *R. camtschaticum* Pall. We identified three unresolved or weakly supported duplications in the CYC2 clade that were shared by the remaining subgenera *Azaleastrum*, *Hymenanthes*, and *Rhododendron* and occurred after their divergence from *R. camtschaticum* (Figure 2, filled red stars). Members of subgenus *Rhododendron* shared a more recent duplication in the CYC2 clade (PP = 0.91; Figure 2, empty red star). The subgenera *Azaleastrum* and *Hymenanthes* also had lineage-specific duplications in the CYC2 clade, as illustrated by *R. simsii* Planch. and *R. delavayi* Franch./*R. williamsianum* Rehder and E.H. Wilson, respectively. Additional lineage-specific duplications were found in subgenus *Rhododendron*, e.g., in *R. edgeworthii* Hook. f. and *R. meliphagidum* J.J.Sm. In the two experimental species, we found five paralogs in *R. beyerickianum* (*RhbCYC2.1*–*RhbCYC2.5*) and four paralogs in *R. taxifolium* (*RhtCYC2.1*–*RhtCYC2.4*) from the CYC2 lineage, which suggests the loss of an *RhtCYC2.5* paralog in *R. taxifolium*. In spite of weaker resolution, we were able to detect additional losses of one paralog in sect. *Schistanthe*, one in *R. meliphagidum*, and two in *R. williamsianum* from the CYC2 lineage. In summary, we identified three main duplications in *Rhododendron* in the CYC2 clade that were shared by subgenera *Azaleastrum*, *Hymenanthes*, and *Rhododendron* and were difficult to resolve. We also uncovered at least seven lineage-specific duplications at the subgeneric or species level and at least five losses at the sectional or species level in the CYC2 clade.

#### 2.1.2. Tandem Gene Duplication and Genetic Variation in *Rhododendron CYC* Orthologs

The paralogous CYC2 clades from subgenera *Azaleastrum* and *Hymenanthes* may be difficult to resolve because these genes appeared to be tandemly duplicated and may be recombining. We recovered three *CYC* orthologs tandemly arranged along chromosome (linkage group (LG)) 11 in *R. williamsianum*, four genes along scaffold3761 in *R. delavayi*, and five genes along chromosome 10 in *R. simsii* (Figure 2, Supplementary Table S2). Therefore, we expect *CYC* orthologs from subgenus *Rhododendron* to also be tandemly duplicated from shared duplications with subgenera *Azaleastrum* and *Hymenanthes*.

Full-length open reading frames (ORFs) of *CYC* orthologs from sampled *Rhododendron* species varied from 762 to 825 bp and consisted of two exons and one intron. Partial coding sequences (CDS) of *CYC* orthologs in the two experimental species, *R. beyerinckianum* and *R. taxifolium,* ranged from 643 to 781 bp (Supplementary Figure S1), and these orthologs were 98–99% identical at the nucleotide level and 96–99% identical at the amino acid level. Paralogs within *R. beyerinckianum* were 87–94% identical in terms of nucleotides and 76–89% identical in terms of amino acids, whereas paralogs within *R. taxifolium* were 90–93% identical in terms of nucleotides and 84–88% identical in terms of amino acids. Among the CYC2 proteins from the experimental species, most of the differences were at the C-terminus (Supplementary Figure S2). In *R. beyerinckianum*, a 5-bp insertion at the 5′ end of *RhbCYC2.3* caused a frameshift mutation that resulted in an early stop codon. Despite this N-terminus truncation, an alternative downstream start site was in use, based on recovered transcripts. *CYC* orthologs from the experimental species did not have the characteristic ECE motif but instead had an ESE, GSE, or GCE amino-acid motif at this location, except for *RhtCYC2.2,* which had a GSD motif (Supplementary Figure S2). Additionally, *Rhododendron CYC* orthologs did not have the conserved LxxLL motif but instead had a IDWLL motif in the the second helix of the TCP domain. In summary, *CYC* orthologs were relatively similar between experimental species *R. beyerinckianum* and *R. taxifolium*, but paralogs of *R. beyerinckianum* showed more variation than those of *R. taxifolium*; we also detected departures from conserved motifs across *Rhododendron CYC* orthologs.

#### 2.1.3. A Shared Duplication in *Rhododendron RAD* Orthologs Arose from an Ericalean Ancestor

*Rhododendron RAD* homologs were analyzed in a phylogenetic context to identify duplications and losses in the RAD2 lineage. We recovered *Rhododendron* orthologs from all three RAD clades (RAD1–3), with RAD2 forming a monophyletic group (PP = 0.59), excluding *Vitis vinifera* L. *RAD* (Figure 3). However, the RAD1 and RAD3 lineages were not recovered as monophyletic groups. Instead, orthologs from the RAD1/RAD3 lineages together formed a monophyletic group (PP = 0.60). From our expanded sampling of *Rhododendron* species and Ericales outgroups in the RAD2 clade, we identified one main duplication in the RAD2 lineage that was shared by *Actinidia* and Ericaceae and likely was present in an ancestor of Ericales (PP = 0.99; Figure 3, red star). No subsequent duplications or losses were found in *Rhododendron RAD* orthologs. We found two paralogs in the RAD2 lineage in both experimental species, *R. beyerinckianum* (*RhbRAD2.1*–*RhbRAD2.2*) and *R. taxifolium* (*RhtRAD2.1*–*RhtRAD2.2*), that were inherited from the duplication observed in an ancestor of Ericales. In summary, one ancient duplication in the RAD2 lineage, in an Ericalean ancestor, resulted in two paralogs in *Rhododendron*, with no subsequent duplications or losses.

#### 2.1.4. Structural and Genetic Variation in *Rhododendron RAD* Orthologs

Full-length ORFs of *RAD* orthologs from sampled *Rhododendron* species varied from 243 to 317 bp. One paralog was intronless due to an early stop codon, consisting of a single exon, whereas the other paralog consisted of two exons and one intron (Supplementary Figure S1). The full-length CDS of *R. beyerinckianum* and *R. taxifolium RAD* orthologs ranged from 246 to 297 bp (Supplementary Figures S1 and S3), and these orthologs were 99–100% identical at the nucleotide level and 97–100% identical at the amino acid level. Paralogs within *R. beyerinckianum* were 81% identical in terms of nucleotides and 78% identical in terms of amino acids. Similarly, paralogs within *R. taxifolium* were 81% identical in terms of nucleotides and 78% identical in terms of amino acids. Differences between paralogs were evenly distributed throughout the MYBI domain and more concentrated in the C-terminal region (Supplementary Figure S3). In summary, *RAD* orthologs were nearly identical between *R. beyerinckianum* and *R. taxifolium*, and paralogs had similar amounts of variation in each species; however, paralogs were quite different due to the loss of an intron and exon in one paralog.

#### 2.1.5. A Shared Duplication in *Rhododendron DIV* Orthologs Arose from an Ericalean Ancestor

Finally, we analyzed *Rhododendron DIV* homologs in a phylogenetic context to identify duplications and losses in the DIV1 lineage. We recovered orthologs from all three DIV clades (DIV1–3/EudiDIV1–3), each with moderate to strong support (PPs = 0.87–1.00) (Figure 4). Expanded sampling of *Rhododendron* species and Ericales outgroups in the EudiDIV1/DIV1 clade identified one main duplication in the EudiDIV1 lineage that again appeared shared by *Actinidia* and Ericaceae and likely was present in an ancestor of Ericales, except one of the paralogs was presumably lost in *Actinidia* (PP = 1.00; Figure 4, red star). No subsequent duplications or losses were identified in *Rhododendron DIV* orthologs. We found two paralogs in the EudiDIV1 lineage for *R. beyerinckianum* (*RhbDIV1.1*–*RhbDIV1.2*) and *R. taxifolium* (*RhtDIV1.1*–*RhtDIV1.2*) that were inherited from the duplication in an Ericalean ancestor (Figure 4). Both *RAD* and *DIV* orthologs underwent one ancient duplication before the origin of *Rhododendron*, potentially in an ancestor of Ericales, with no recent duplications or losses, and preceded duplications and losses of *CYC* orthologs in *Rhododendron*.

#### 2.1.6. Genetic Variation in *Rhododendron DIV* Orthologs

Full-length ORFs of *DIV* orthologs from sampled *Rhododendron* species varied from 855 to 909 bp and consisted of two exons and one intron (Supplementary Figures S1 and S4). The full-length CDS of *R. beyerinckianum* and *R. taxifolium DIV* orthologs ranged from 855 to 888 bp, and these orthologs were 99–100% identical at the nucleotide level and 99% identical at the amino acid level. *R. beyerinckianum* paralogs were 74% identical in terms of nucleotides and 66% identical in terms of amino acids. Similarly, *R. taxifolium* paralogs were 74% identical in terms of nucleotides and 66% identical in terms of amino acids. The *RhbDIV1.2/RhtDIV1.2* paralogs contained a mutation that coded for an early stop codon, presumably resulting in a protein that was 11 amino acids shorter at the C-terminus than RhbDIV1.1/RhtDIV1.1 (Supplementary Figure S4). Most of the amino acid differences between paralogs occurred in the C-terminus, downstream from the MYBI and MYBII domains. In summary, *DIV* orthologs were nearly identical between *R. beyerinckianum* and *R. taxifolium*, and paralogs had similar amounts of variation in each species; however, paralogs were quite variable outside of the conserved MYB domains.

### 2.2. Comparative Expression Analyses of Flower Symmetry Genes in Rhododendron

**Figure 5:**
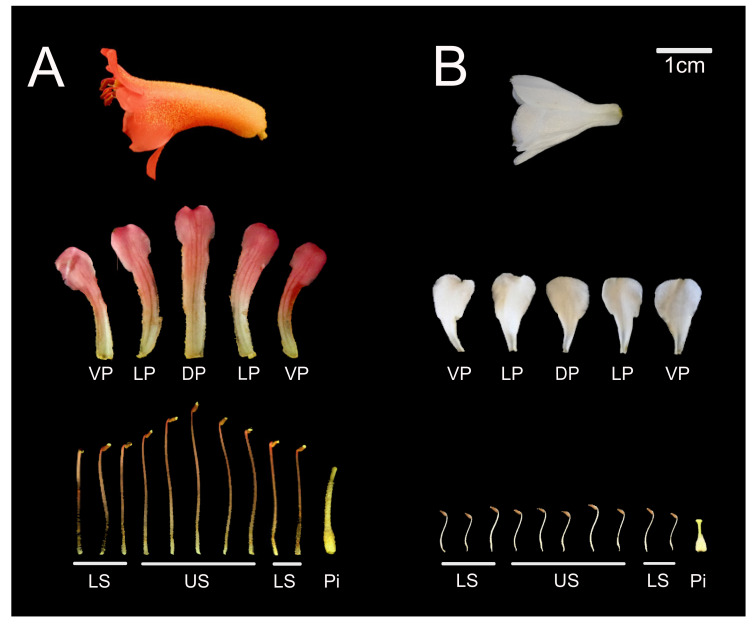
Morphology of open flowers in two *Rhododendron* species with different floral symmetries. (**A**) Bilateral flower of *R. beyerinckianum*. (**B**) Radial flower of *R. taxifolium*. Whole flowers are shown with the dorsal side toward the top. Dissected floral organs are shown below each flower: dorsal petal (DP), lateral petals (LP), ventral petals (VP), upper stamens (US), lower stamens (LS), and pistil (Pi).

We chose two *Rhododendron* species with different floral symmetries from sister clades [55], *R. beyerinckianum* and *R. taxifolium*, to compare expression of flower symmetry genes. Bilateral symmetry in *R. beyerinckianum* flowers results from differential growth across all floral organs. The corolla tube is curved adaxially, upper petals (the dorsal petal in particular) and stamens are longer than lower petals and stamens, and the style is curved downward at maturity (Figure 5). In contrast, *R. taxifolium* flowers are more radial, without corolla tube curvature, exhibiting petals and stamens subequal in length (the latter alternating in length) and a style that is slightly curved downward at maturity (Figure 5). Therefore, we sought to examine whether distinct floral morphologies correlated with changes in candidate gene expression in the two species by reverse transcription-PCR (RT-PCR) in dissected floral organs (petals, stamens, and pistils). We first examined expression patterns across flower development to determine whether differential expression occurred among paralogs across developmental stages and to select the most appropriate stage for comparing across floral organs.

#### 2.2.1. Uniform or Increasing Expression of *CYC* Orthologs across *Rhododendron* Flower Development

**Figure 6:**
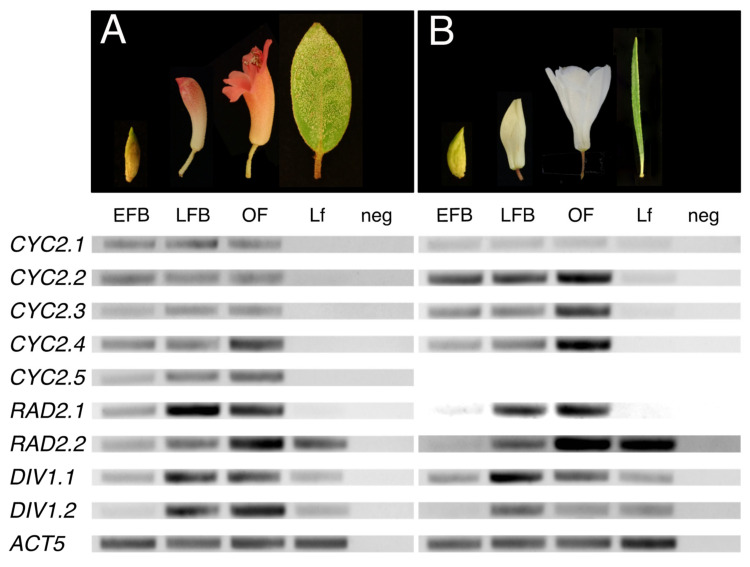
Gene expression of *CYCLOIDEA* (*CYC*), *RADIALIS* (*RAD*), and *DIVARICATA* (*DIV*) orthologs across flower development in two *Rhododendron* species with different floral symmetries. Gene expression shown by RT-PCR. Floral developmental stages sampled are early floral bud (EFB), late floral bud (LFB), and open flower (OF). Vegetative (Lf) and negative (neg) controls are also shown. *ACTIN5* (*ACT5*) is used as a reference gene. (**A**) Bilaterally symmetric *R. beyerinckianum*. (**B**) Radially symmetric *R. taxifolium*.

We examined gene expression patterns ontogenetically using RT-PCR across leaf and floral developmental stages of *R. beyerinckianum* and *R. taxifolium* (Figure 6, Supplementary Figure S5). In *R. taxifolium*, four paralogs from the CYC2 lineage were expressed throughout flower development, from early floral buds to open flowers. Three of the paralogs (*RhtCYC2.2*-*RhtCYC2.4*) showed similar expression patterns consisting of increasing expression from early to late floral stages. The fourth paralog *RhtCYC2.1* showed less expression than the other three and was consistent across all floral developmental stages. Expression in leaves was barely detectable for *RhtCYC2.1*–*RhtCYC2.3* or lacking for *RhtCYC2.4*. Five paralogs from the CYC2 lineage were expressed in *R. beyerinckianum* flowers across all developmental stages. We observed similar expression levels across all floral developmental stages for three of the paralogs, *RhbCYC2.1*–*RhbCYC2.3*. For *RhbCYC2.4*, expression increased from early to late floral stages, but, for *RhbCYC2.5*, expression patterns were inconsistent across replicates. In contrast to *R. taxifolium*, none of the *R. beyerinckianum* paralogs showed expression in leaves. Comparing both species, *RhbCYC2.1* was expressed at similar levels to other paralogs, whereas *RhtCYC2.1* was expressed at lower levels. Additionally, *RhtCYC2.5* was apparently lost in *R. taxifolium*, as we did not recover it through PCR nor transcriptome assembly. Despite differences in expression among paralogs, most *CYC* orthologs showed increasing or uniform expression across floral developmental stages in *Rhododendron* species with different floral symmetries, with barely detectable to no expression in leaves.

#### 2.2.2. Increasing Expression of *RAD* Orthologs across *Rhododendron* Flower Development

Unlike *CYC* orthologs, *RAD* orthologs had less differential expression between paralogs across floral stages in the two experimental species (Figure 6, Supplementary Figure S5). We observed similar expression patterns between paralogs in *R. beyerinckianum* as in *R. taxifolium*. Both paralogs from the RAD2 lineage had higher expression in late compared to early floral stages. Differential expression between the two paralogs consisted of *RhbRAD2.2/RhtRAD2.2* showing strong expression in leaves, whereas *RhbRAD2.1/RhtRAD2.1* was barely detectable. Taken together, all *RAD* orthologs showed increasing expression during flower development in *Rhododendron* species with different floral symmetries, with differential paralog expression occurring mainly in leaves.

#### 2.2.3. Increasing Expression of *DIV* Orthologs across *Rhododendron* Flower Development

Differential expression between paralogs from the DIV1 lineage occurred within and between the experimental species (Figure 6, Supplementary Figure S5). Similar to *CYC* and *RAD* orthologs, we observed increasing expression of *DIV* orthologs from early to late floral stages in both species. However, in *R. taxifolium*, *RhtDIV1.1* appeared to have higher expression than *RhtDIV1.2* across floral stages, whereas, in *R. beyerinckianum*, *RhbDIV1.2* appeared to have higher expression than *RhbDIV1.1* across floral stages. Both paralogs showed moderate and comparable expression in leaves for both species. Taken together, paralogs from the DIV1 lineage were differentially expressed in both species; one paralog was expressed more than the other across floral development. Despite differences in expression between paralogs, all *DIV* orthologs showed increasing expression across floral developmental stages in *Rhododendron* species with different floral symmetries, with moderate expression in leaves.

#### 2.2.4. Dorsally Restricted Expression of *CYC* Orthologs in Bilateral Flowers versus Ubiquitous Expression in Radial Flowers

**Figure 7:**
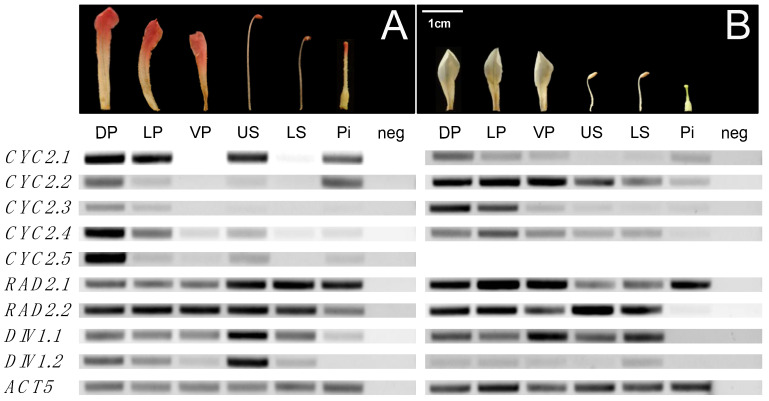
Gene expression of *CYCLOIDEA* (*CYC*), *RADIALIS* (*RAD*), and *DIVARICATA* (*DIV*) orthologs across floral organs in two *Rhododendron* species with different floral symmetries. Gene expression shown by RT-PCR. Floral organs sampled from late floral buds are dorsal petals (DP), lateral petals (LP), ventral petals (VP), upper stamens (US), lower stamens (LS), and pistils (Pi). Negative (neg) controls are also shown. *ACTIN5* (*ACT5*) is used as a reference gene. (**A**) Bilaterally symmetric *R. beyerinckianum*. (**B**) Radially symmetric *R. taxifolium*.

Expression of flower symmetry candidate genes increased from early to late floral stages in *R. beyerinckianum* and *R. taxifolium*. Therefore, we selected the intermediate stage of late floral buds to compare gene expression across floral organs. Paralogs from the CYC2 lineage were differentially expressed across floral organs within and between species (Figure 7, Supplementary Figure S6). In *R. taxifolium*, *RhtCYC2.1* showed low levels of expression primarily across petals and little to no expression in stamens and pistils. *RhtCYC2.2* showed the highest expression across all organs and paralogs. *RhtCYC2.3* was expressed at higher levels in the upper petals (dorsal and lateral petals) than in the ventral petals, stamens, and pistils. *RhtCYC2.4* was expressed across all floral organs but with decreasing expression in the stamens and pistils. In *R. beyerinckianum*, we observed more striking differential expression across floral organs and paralogs. All five paralogs showed higher expression in dorsal and lateral petals than ventral petals and higher expression in upper stamens than lower stamens; most of these paralogs were barely detectable in ventral petals and stamens. *RhbCYC2.1* exhibited the highest expression in dorsal petals, lateral petals, and upper stamens among paralogs. *RhbCYC2.5* showed the most restricted dorsal expression of all paralogs, with its highest expression in dorsal petals, while *RhbCYC2.4* showed the least restricted expression, decreasing from dorsal to ventral organs. *RhbCYC2.3* showed the lowest expression overall, and *RhbCYC2.1* was highest for pistils. This is in contrast to *RhtCYC2.1*, which had much lower expression in pistils of *R. taxifolium*. Taken together, we observed differential expression among paralogs from the CYC2 lineage both within and between the two species. Despite within-species differences, expression of *CYC* orthologs was more uniform across floral organs in the radial flowers of *R. taxifolium,* whereas it was more restricted to the dorsal region in bilateral flowers of *R. beyerinckianum*.

#### 2.2.5. Ubiquitous Expression of *RAD* Orthologs in Bilateral and Radial *Rhododendron* Flowers

*Rhododendron* paralogs from the RAD2 lineage had relatively similar expression patterns across floral organs in both species, with minor differences (Figure 7, Supplementary Figure S6). In *R. taxifolium*, both paralogs were expressed at similar levels across floral organs with the exception of *RhtRAD2.2*, which was barely or not expressed in pistils. In *R. beyerinckianum*, we observed similar expression patterns across floral organs in both paralogs, where *RhbRAD2.2* had lower expression in pistils than *RhbRAD2.1*. Taken together, *RAD* orthologs were uniformly expressed across floral organs in both species; we did not observe pronounced differential expression among *Rhododendron RAD* orthologs within or between species with different floral symmetries.

#### 2.2.6. Uniform Expression of *DIV* Orthologs in Bilateral and Radial *Rhododendron* Flowers

*Rhododendron DIV* orthologs showed more differential expression between paralogs and species than *RAD* orthologs. In *R. taxifolium*, *RhtDIV1.1* was expressed at higher levels across floral organs than *RhtDIV1.2* (Figure 7, Supplementary Figure S6). However, we did not observe strong differences in expression between the two paralogs in *R. beyerinckianum*. Both paralogs were expressed at similar levels across floral organs with the exception of *RhbDIV1.2*, which had lower expression in the pistil than *RhbDIV1.1*. Taken together, we observed differential expression between the two paralogs from the DIV1 lineage in radially symmetric *R. taxifolium* but not in bilaterally symmetric *R. beyerinckianum*. Despite differences in expression between paralogs, each *Rhododendron DIV* ortholog was uniformly expressed across floral organs in species with different floral symmetries.

## 3. Discussion

**Figure 8:**
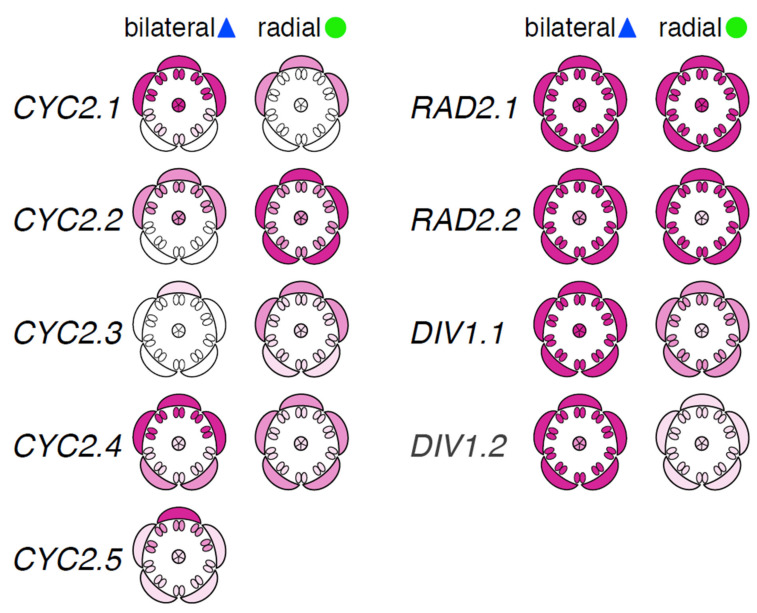
Summary of gene expression patterns for *CYCLOIDEA* (*CYC*), *RADIALIS* (*RAD*), and *DIVARICATA* (*DIV*) orthologs across floral organs in two *Rhododendron* species with different floral symmetries. Gene expression from RT-PCR was quantified and averaged across three biological replicates for each species, then visualized on floral diagrams of *Rhododendron* via cross-section. Floral diagrams show the five petals, ten stamens, and central pistil in flowers of the two species. Color intensity denotes expression level; white denotes no expression. Bilateral, blue triangle = bilaterally symmetric *R. beyerinckianum*; radial, green circle = radially symmetric *R. taxifolium*. Note: A *CYC2.5* ortholog could not be found in *R. taxifolium*.

Our aim was to assess the genetic underpinnings of flower symmetry variation in *Rhododendron*, an early diverging lineage of asterids, to address the depth of conservation of this genetic network within asterids. To that end, we investigated the duplication history and expression pattern of orthologs of the candidate genes *CYC*, *RAD*, and *DIV* in species with radially and bilaterally symmetric flowers. We found extensive evidence of duplication and loss in *CYC* orthologs among *Rhododendron* species, resulting in five paralogs in the bilaterally symmetric species *R. beyerinckianum* and four paralogs in the radially symmetric species *R. taxifolium*. In contrast to *CYC* orthologs, we did not find evidence of duplication nor loss in *RAD* or *DIV* orthologs in *Rhododendron*; the genus inherited two paralogs for each of these candidate genes from an ancient duplication present in an Ericalean ancestor. On the one hand, we observed dorsally restricted expression among paralogs from the CYC2 lineage in upper petals and upper stamens and strong expression in pistils for one paralog in species with bilateral flower symmetry. On the other hand, we observed more uniform expression across petals and stamens among paralogs from the CYC2 lineage and weak expression in pistils in species with radial flower symmetry (Figure 8). In contrast to *CYC* orthologs, we did not observe differential expression patterns in dorsal versus ventral organs of bilateral flowers across *RAD* and *DIV* orthologs. Both paralogs from the RAD2 and DIV1 lineages were uniformly expressed across petals and stamens in both species (Figure 8). Moreover, *CYC* orthologs appeared flower-specific, whereas *RAD* and *DIV* orthologs were expressed in both reproductive and vegetative tissues across species. Our findings suggest that *Rhododendron CYC* orthologs have undergone gene duplications and losses and changes in expression patterns comparable to those observed in other bilaterally or radially symmetric asterids, with certain exceptions for *RAD* and *DIV* orthologs. One possible explanation for our results is that *RAD* orthologs were regulated differently in *Rhododendron* (e.g., not directly by *CYC* orthologs) and that they became involved in flower symmetry in later-diverging asterids.

### 3.1. Evolution of *Rhododendron CYC* Orthologs Compared to Other Asterids

#### 3.1.1. Tandem Gene Duplication Creates Diversity in *Rhododendron CYC* Orthologs

We observed extensive gene duplication in *Rhododendron CYC* orthologs that was shared by subgenera or that was lineage-specific in certain taxa. Based on genomic mining of *CYC* orthologs from *R. delavayi* [49], *R. simsii* [53], and *R. williamsianum* [51], we determined that these duplications resulted from tandem gene duplication, as duplicates occurred sequentially along a scaffold or chromosome (Supplementary Table S3). In fact, tandem and proximal duplications have contributed to approximately 26% of gene family expansion in *R. simsii* and are associated with stronger positive selection [53]. Both whole genome duplication (WGD) and tandem duplication have contributed to the evolution of TCP genes in *Antirrhinum*; the Plantaginaceae-specific WGD is responsible for the *CYC*/*DICH* paralogs found in this species [11]. Asterales has undergone extensive gene duplication in *CYC* orthologs that resulted in part from tandem duplication. In *Helianthus*, five *CYC* orthologs have evolved from four duplication events, with three of them tandemly located on LG9, and the other two translocated to two other LGs [58]. Tandemly arrayed, duplicated genes tend to occur in high recombination regions (“hotspots”) and are likely caused by unequal crossing over [59]. Therefore, *CYC* orthologs in *Rhododendron* are likely in such hotspots, making phylogenetic inference difficult due to recombination. Given that selection is considered more efficient in high recombination regions [59], these hotspots likely provided the raw material for selection to act upon to contribute to the floral diversity observed in *Rhododendron*.

#### 3.1.2. Conserved Patterns of Dorsally Restricted Expression of *CYC* Orthologs in Bilateral *Rhododendron* Flowers

In the bilaterally symmetric *R. beyerinckianum*, differential expression was observed across petals and stamens in all five paralogs from the CYC2 lineage, where expression was higher in dorsal and lateral petals and upper stamens and often barely detectable in ventral petals and lower stamens (Figure 8). *RhbCYC2.3* showed the weakest expression among paralogs, which was likely due to the truncated protein at the N-terminus. Subfunctionalization was likely in *RhbCYC2.5,* since it had the most restricted dorsal expression among paralogs, with the highest expression in the dorsal petal across organs. This differential expression pattern likely contributed to the dorsal petal being the longest in *R. beyerinckianum* (Figure 5). Besides *Antirrhinum*, similar patterns have been observed in other bilaterally symmetric species in asterids, where one *CYC* ortholog is more dorsally restricted than the other(s) [31,60,61,62,63,64,65]. Therefore, in *Rhododendron,* we see conserved patterns of differential expression of *CYC* orthologs in dorsal versus ventral regions of the flower that are associated with bilateral symmetry.

#### 3.1.3. Differential Expression of *CYC* Orthologs in Bilateral *Rhododendron* Flowers Is Associated with Differential Growth in Petals and Stamens

*CYC* orthologs can promote or repress growth of different floral organs in a variety of lineages through the control of genes involved in cell proliferation and expansion [4,14]. However, unlike *Antirrhinum*, where *CYC* expression correlates with repression of growth in the dorsal region of the flower early in development [6], *R. beyerinckianum* expression correlates with promotion of growth in the dorsal region of the flower, where longer dorsal and lateral organs have higher gene expression than the shorter ventral organs (Figure 5 and Figure 8). *CYC* orthologs are also known to promote petal growth in Asterales [66] and in *Antirrhinum* at later floral stages [6]. However, to our knowledge, no other studies have reported the promotion of growth in stamens associated with the expression of *CYC* orthologs as observed here for *R. beyerinckianum*.

#### 3.1.4. *CYC* Orthologs Are Associated with Asymmetric Organ Growth in Bilateral *Rhododendron* Flowers

We observed curvature of lateral and ventral petals in *R. beyerinckianum*, which contributed to an adaxially curved corolla tube and bilateral symmetry in this species (Figure 5). In *Antirrhinum*, *CYC* expression promotes dorsal identity in the dorsal petals, contributing to overall floral bilateral symmetry, whereas *DICH* expression promotes asymmetry within each dorsal petal [8]. In another bilaterally symmetric asterid, *Sinningia* (Lamiales), the *CYC* ortholog (*SsCYC*) is associated with different morphologies in the dorsal, lateral, and ventral petals: petal outward curvature, midrib asymmetry, and dilation of the tube, respectively [67]. Therefore, *CYC* orthologs may also be acting differentially within an organ in *Rhododendron*, potentially contributing to asymmetric growth of lateral and ventral petals in *R. beyerinckianum*.

Styles are often curved in bilaterally symmetric *Rhododendron* species, with the style usually curved downward in adaxially curved corollas (Figure 5) [47]. In *R. beyerinckianum*, all paralogs from the CYC2 lineage were expressed in pistils, and *RhbCYC2.1* showed the strongest expression, in contrast to *RhtCYC2.1*, which had barely detectable expression in the short pistils of *R. taxifolium* (Figure 8). This difference in expression in pistils suggests that *CYC* orthologs may also promote asymmetric growth in the pistil that contributes to style curvature in *R. beyerinckianum*. While expression of *CYC* orthologs in pistils has been observed in other asterids, it has not been associated with differences in floral symmetry [31,33,37,58,68,69]. Therefore, *CYC* orthologs may show a novel function in asymmetric growth of the pistil in *Rhododendron*.

#### 3.1.5. Multiple Conserved Expression Patterns of *CYC* Orthologs in Radial *Rhododendron* Flowers

We observed several differences in gene expression in *R. taxifolium* compared to *R. beyerinckianum* that likely contributed to radial symmetry in the former (Figure 8). First, the ortholog of *RhbCYC2.5*, which showed the dorsal-most restriction of expression in *R. beyerinckianum*, has been lost in *R. taxifolium.* Second, the ortholog of *RhbCYC2.1*, which exhibited the highest expression in dorsal and lateral petals and upper stamens of *R. beyerinckianum*, showed the weakest expression in *R. taxifolium* and appeared petal specific. Finally, two paralogs (*RhtCYC2.2* and *RhtCYC2.4*) were uniformly expressed across all organ types. Ubiquitous expression of *CYC* orthologs across petals and/or stamens has also been associated with radially symmetric asterids [33,37,62,70]. In other asterids, loss of expression or function of *CYC* orthologs is associated with radial symmetry [30,32,33,34,35,36]. Therefore, we see multiple conserved patterns in *Rhododendron CYC* orthologs in a radially symmetric species: ubiquitous expression across floral organs, reduced expression, and gene loss.

### 3.2. Evolution of Rhododendron RAD and DIV Orthologs Compared to Other Asterids

#### 3.2.1. Divergent Patterns of Gene Structure, Duplication, and Expression of *Rhododendron RAD* Orthologs

In contrast to *CYC* orthologs, *Rhododendron RAD* and *DIV* orthologs reside on different scaffolds or chromosomes (Supplementary Table S3) and likely resulted from a WGD in an Ericalean ancestor [51,53,71]. These genes have not duplicated or undergone loss as in the *Rhododendron* CYC2 lineage. Moreover, one of the *Rhododendron RAD* orthologs (*RhbRAD2.2/RhtRAD2.2*) has a different structure from that reported in other asterids and rosids. *Antirrhinum* and *Arabidopsis RAD* orthologs have two exons with coding regions of similar length and an intron in a conserved position [38], whereas this *Rhododendron* ortholog has a single exon, suggesting loss of the regulatory intron and second exon. However, these differences in gene structure between the two *Rhododendron* paralogs from the RAD2 lineage do not appear to affect gene expression (Figure 8). Thus, *Rhododendron RAD* orthologs diverge from other asterids in their gene structure and pattern of gene duplication.

*RAD* is positively regulated by *CYC* in *Antirrhinum* and suppresses DIV activity from dorsal regions of the flower [14,15]. Dorsal expression of *RAD* orthologs corresponds with expression of *CYC* orthologs across bilaterally symmetric asterid flowers [27,30,31,32]. However, we did not observe similar expression patterns between *RAD* orthologs and *CYC* orthologs in bilaterally symmetric flowers of *Rhododendron*. Instead, we see ubiquitous expression of *RAD* orthologs across floral organs regardless of floral symmetry (Figure 8). This result implies that *RAD* orthologs are unlikely to be directly regulated by *CYC* orthologs in *Rhododendron* and that *RAD* orthologs may not contribute to floral symmetry through interactions with *DIV* orthologs in the same manner as in other asterids. Consistent with our findings, expression of *RAD* orthologs does not appear to be regulated by *CYC* orthologs outside of *Antirrhinum*, as the timing of expression between these two genes in petals is different in early diverging Lamiales [37]. Moreover, considering that *RAD* orthologs are not expressed in leaves in Lamiales [31,37], evidence presented here for *Rhododendron RAD* leaf expression suggests a different, non-flower specific function for these genes.

#### 3.2.2. Conserved Ubiquitous Patterns of Expression of *DIV* Orthologs in *Rhododendron* Flowers

Both paralogs from the DIV1 lineage are uniformly expressed across all floral organs in both bilaterally and radially symmetric flowers of *Rhododendron* (Figure 8). We did not expect to see differences in expression across floral organs in *Rhododendron* given that *DIV* is transcribed in all floral organs of *Antirrhinum* and is inhibited post-translationally in the dorsal region by *RAD* [12,13,15]. In *Antirrhinum*, *DIV* and another MYB gene, *DIV-AND-RAD-INTERACTING-FACTOR* (*DRIF*), display overlapping expression patterns in the corolla, forming heterodimers that activate downstream ventral identity targets and upregulate *DIV* transcription [15]. RAD acts as a small interfering peptide and competes with DIV to interact with DRIF in the dorsal region of flowers [25]. Therefore, DIV-DRIF heterodimerization and *DIV* transcription are inhibited post-translationally in the dorsal region, where RAD binds to DRIF [15]. Given their overlapping expression patterns, *Rhododendron DIV* orthologs may be inhibited post-translationally by *RAD* orthologs, but further experiments are needed to determine this. The expression of *DIV* orthologs throughout all floral organs appears to be shared across bilaterally and radially symmetric asterids [26,30,34,35]. Since *DIV* has been shown to be post-translationally regulated, this complicates inferences of function from expression patterns. However, we predicted that paralogs from the DIV1 lineage would have evolved different expression patterns, and we observed this to some extent in *R. taxifolium* (Figure 8). Similar expression patterns of paralogs from the DIV1 lineage across petals, stamens, and carpels have also been observed in the asterid *Bournea* (Lamiales) [30]. Furthermore, we found expression of *DIV* orthologs in *Rhododendron* leaves, which is consistent with expression patterns found in other asterids [26,66] and suggests that these genes are not flower-specific.

### 3.3. Future Directions in Rhododendron Floral Symmetry Research

In this study, we sampled flowers from two derived species of tropical rhododendrons with bilateral and radial symmetry, respectively. While our study is a first step toward understanding the genetics of flower symmetry in this group, these two species clearly cannot capture the entire variation in floral symmetry within the section (*Schistanthe*) nor reconstruct the ancestral state. Increased sampling, to include representatives from other clades, should improve the reconstruction of the evolution of gene expression in *Rhododendron CYC*, *RAD*, and *DIV* orthologs. As shown in other groups, e.g., Lamiales [32], radial symmetry can be derived from bilateral symmetry in different ways developmentally, including changes in merosity or in other flower symmetry genes. Therefore, future studies in *Rhododendron* should examine other transitions in symmetry outside of sect. *Schistanthe* to determine whether the genetic and developmental mechanisms involved are similar or different across the genus.

Now that we have a theoretical model for *CYC* orthologs as potentially promoting differential growth of floral organs in *Rhododendron*, we can use this to predict flower symmetry candidate gene expression in other bilaterally symmetric flowers in the genus. The ancestral *Rhododendron* presumably had a bilaterally symmetric corolla with abaxially positioned stamens and style (Figure 1F), similar to flowers of *R. camtschaticum* [46]. In this type of ancestral bilateral symmetry, we predict that differential expression of *CYC* orthologs in ventral stamens and styles promotes longer lower stamens and upward style curvature. Transitions from these temperate, bilaterally symmetric species to radially symmetric flowers in subgenus *Azaleastrum* (e.g., *R. albiflorum* Hook.) and in subgenus *Rhododendron* (e.g., former *Ledum*) may have recruited *CYC* orthologs in similar or different ways to tropically derived radial symmetry. In contrast, transitions to radially symmetric flowers due to merosity changes, in the former *Menziesia* (i.e., reductions to 4-merous flowers) and in subgenera *Hymenanthes* (e.g., *R. williamsianum*) and *Rhododendron* (e.g., *R. konori* Becc.) (i.e., increases in petal lobe number; Figure 1C), may have deployed similar *CYC* genetic mechanisms as *Plantago* [33]. Finally, other tropical species that exhibit bilaterally symmetric flowers due to abaxial curvature of the corolla tube, e.g., *R. tuba* (Figure 1D), may have inverse expression patterns from those of *R. beyerinckianum*. In these bilaterally symmetric flowers, we predict that *CYC* orthologs will be differentially expressed in longer ventral petals, contributing to the abaxial curvature of the corolla tube. Bilaterally symmetric corollas in temperate species can also be due to differences in petal connation (Figure 1A), which likely involves different genetic mechanisms than *CYC* orthologs. Thus, the genus *Rhododendron* has a remarkable amount of variation in floral symmetry, which will require further study from a genetic and developmental perspective to fully understand the extent of evolutionary innovation in this group.

## 4. Materials and Methods

### 4.1. Data Mining for CYC, RAD, and DIV Homologs in Rhododendron

We obtained homologs of flower symmetry candidate genes from the following publicly available *Rhododendron* genomes: *R. delavayi* [49], *R. simsii* [53], and *R. williamsianum* [51] (Supplementary Table S2). *A. majus CYC, DICH*, *RAD*, and *DIV* sequences (Supplementary Table S2) were used to search these genomes using blastn, with default settings in BLAST+ v2.6.0 [72,73].

### 4.2. Isolation of Rhododendron CYC, RAD, and DIV Orthologs

To further investigate the duplication history of *CYC*, *RAD*, and *DIV* orthologs within *Rhododendron*, we sampled five additional species from two of the five subgenera [44] (Supplementary Table S1). Additionally, three closely related Ericaceae outgroups were sampled for in-house molecular isolation of these genes (Supplementary Table S1). The following *Rhododendron* subgenera were sampled: *Azaleastrum* (*R. simsii*), *Hymenanthes* (*R. delavayi*, *R. williamsianum*), *Rhododendron* (*R. beyerinckianum*, *R. edgeworthii*, *R. meliphagidum*, *R. taxifolium*), and *Therorhodion* (*R. camtschaticum*). DNA was extracted from leaves or floral buds using the DNeasy^®^ Plant Mini Kit (QIAGEN^®^, Valencia, CA, USA) with a modified protocol as described in Soza et al. [51].

Primers for *CYC*, *RAD*, and *DIV* orthologs were initially designed from the *R. williamsianum* genome. We designed degenerate primers to capture all *CYC* orthologs near the start and stop codons of the gene and further refined these after procuring additional *Rhododendron* sequences (Supplementary Table S3). We also designed degenerate primers in the conserved TCP and R domains to capture *CYC* orthologs from outgroups (Supplementary Table S3). We designed paralog-specific primers for *RAD* and *DIV* orthologs (Supplementary Table S3). Primers were designed to amplify the first exon of *RAD* orthologs, whereas primers amplified the first exon and the MYBI domain in the second exon of *DIV* orthologs. We used the OligoAnalyzer Tool (Integrated DNA Technologies, Coralville, IA, USA) to confirm primer pair compatibility before initiating PCR.

We used Phusion^®^ High-Fidelity DNA Polymerase (New England Biolabs^®^ Inc., Ipswich, MA, USA) in 20-µL reactions, according to manufacturer’s recommendations for PCR, with 1 µL of 1:10 template DNA. PCR was conducted in T100^™^ thermal cyclers (Bio-Rad Laboratories, Inc., Hercules, CA, USA) with an initial denaturation at 98 °C for 30 s; followed by 35 cycles of denaturation at 98 °C for 10 s, annealing at 52–67 °C for 30 s, and extension at 72 °C for 30 s or 60 s; with a final extension at 72 °C for 10 min (Supplementary Table S3). One ortholog of *DIV* and one ortholog of *RAD* were readily sequenced by direct sequencing, but the other *RAD* and *DIV* orthologs as well as all *CYC* orthologs required cloning. PCR products were cleaned using a MinElute^®^ PCR Purification Kit or Gel Extraction Kit (QIAGEN^®^, Valencia, CA, USA), then A-tailed with a Taq (Promega Inc., Fitchburg, WI, USA) or Klenow Fragment (3′->5′ exo-) (New England Biolabs^®^ Inc., Ipswich, MA, USA) and ligated into the pCR^™^2.1-TOPO^®^ vector (Invitrogen^™^, Life Technologies^™^, Carlsbad, CA, USA), or ligated directly into the pCR^™^4Blunt-TOPO^®^ vector (Invitrogen^™^, Life Technologies^™^, Carlsbad, CA, USA) without A-tailing. Plasmids were transformed into NEB^®^ 5-alpha Competent *E. coli* (High Efficiency) (New England Biolabs^®^ Inc., Ipswich, MA, USA) or One Shot^™^ TOP10 chemically competent *E. coli* cells (Invitrogen^™^, Life Technologies^™^, Carlsbad, CA, USA), following the manufacturer’s protocol. Transformants were plated onto selective agar media, and 12 (*RAD*/*DIV* orthologs) or 36–78 (*CYC* orthologs) clones from each reaction were screened by PCR using M13 primers (Supplementary Table S3). PCR was performed using an initial incubation at 94 °C for 10 min, followed by an initial denaturation at 98 °C for 30 s, then 30 cycles of denaturation at 98 °C for 10 s, annealing at 55 °C for 30s, and extension at 72 °C for 30 s or 60 s, with a final extension at 72 °C for 10 min. PCR fragments of the expected size, as visualized on agarose gels, were treated with ExoSAP-IT^®^ (Affymetrix, Inc., Cleveland, OH, USA) and sequenced in-house on a 3130*xl* Genetic Analyzer (Applied Biosystems^®^, Life Technologies^™^, Carlsbad, CA, USA) or by GENEWIZ (Seattle, WA, USA) with T3/T7 or M13 primers.

We also generated in-house transcriptomes for *R. beyerinckianum* and *R. taxifolium* [54] from pooled tissue (early floral buds, late floral buds, open flowers, fruits, leaves, and terminal vegetative buds) to verify *CYC*, *RAD*, and *DIV* orthologs that were obtained from the PCR methods above and to elucidate gene structure. Only partial transcripts of *CYC* orthologs were obtained from the assemblies, but complete transcripts of *RAD* and *DIV* orthologs were used for gene structure inferences. Nucleotide and amino acid sequences were imported into Jalview v2.11.1.0 [74] for visualization and to calculate identicality.

### 4.3. Phylogenetic Analyses of Rhododendron CYC, RAD, and DIV Orthologs

Sequences obtained from PCR were assembled in Sequencher v5.4.6 (Gene Codes Corporation, Ann Arbor, MI, USA) and aligned in Mesquite v3.6 [75], excluding primer sequences. We manually detected and removed recombinant sequences among clones from *Rhododendron CYC* orthologs. We confirmed the majority of recombinants with at least one method in RDP4 [76] using a full exploratory recombination scan to include RDP [77], GENECONV [78], MaxChi [79,80], BootScan [81,82], and SiScan [83] across clonal sequences for each taxon. Analyses were run under the default settings as linear sequences and disentangling overlapping signals. We did not observe PCR-mediated recombination among *RAD* and *DIV* orthologs.

An in-house transcriptome of *R. camtschaticum* [54] was used to verify the single PCR-based *CYC* ortholog found in this species. We used *A. majus CYC* and *DICH* sequences (Supplementary Table S2) and consensus sequences from clones generated above to search the *R. camtschaticum* transcriptome with blastn under default settings in BLAST+. After preliminary phylogenetic analyses of all clonal and transcript sequences, consensus sequences representing each monophyletic group of sequences from a taxon were generated inclusively, including SNPs if they occurred in more than one clone, for use in the final alignment.

Orthologs of flower symmetry candidate genes were obtained from other publicly available Ericales genomes for phylogenetic analyses: *Actinidia chinensis* Planch. [52] and *Vaccinium corymbosum* L. [50] (Supplementary Table S2). We used *R. delavayi CYC*, *RAD*, and *DIV* orthologs (Supplementary Table S2) to search genomes with blastn under default settings in BLAST+. We also included sequences of *A. majus*, *Arabidopsis thaliana* (L.) Heynh., *Solanum lycopersicum* L., and *V. vinifera* (Supplementary Table S2) from the CYC-, DIV-, and RAD1–3 lineages [28,84] to identify *CYC*, *DIV*, and *RAD* orthologs in *Rhododendron*. Only the conserved TCP and R domains were used for the CYC1, CYC3, and non-ericaceous CYC2 lineages, as other regions were unalignable across these sequences.

We reconstructed the phylogeny for each floral symmetry gene dataset using Bayesian analyses on the nucleotide alignment. Ambiguously aligned regions were excluded from analyses. The model of evolution was determined by jModelTest v2.1.10 [85,86] via the CIPRES Science Gateway v3.3 [87]. The models selected under the Akaike Information Criterion [88] were the transversion model (TVM), with a proportion of invariant sites and a gamma distribution of rate heterogeneity, for the CYC dataset and the general time reversible (GTR) model, with a proportion of invariant sites and a gamma distribution of rate heterogeneity, for the RAD and DIV datasets. We specified *Aquilegia caerulea* E.James as the outgroup for the CYC dataset [84] and *Magnolia grandiflora* L. as the outgroup for both RAD and DIV datasets [28] (Supplementary Table S2). Bayesian analyses were conducted in MrBayes v3.2.7a [89,90] via the CIPRES Science Gateway. We used default priors of no prior knowledge for the parameters of the model. Bayesian analyses were conducted with three independent Markov Chain Monte Carlo [91] analyses of 10–60 million generations each. Metropolis coupling for each analysis was conducted under the default settings. Convergence was determined when the average standard deviation of split frequencies remained less than 0.01. The first 16% of CYC trees, 57% of RAD trees, and 13% of DIV trees were discarded before convergence. The remaining trees from each run were pooled to construct a 50% majority-rule consensus tree to obtain PPs, which was then visualized with FigTree v1.4.3 [92].

### 4.4. RNA Sample Collection

To investigate the expression of flower symmetry candidate genes, we collected leaves and floral tissue at three stages of development from two closely related *Rhododendron* species in sect. *Schistanthe*. All plant material was collected from the Rhododendron Species Botanical Garden, Federal Way, WA, USA. We collected two species from sister clades identified in Soza et al. [55]: *R. beyerinckianum* and *R. taxifolium* (Supplementary Table S1). *R. beyerinckianum* represents a derived form of bilateral symmetry in sect. *Schistanthe*, with curved floral tubes and longer dorsal than ventral petals [55] (Supplementary Figure S5), whereas *R. taxifolium* represents a derived form of radial symmetry [55] (Supplementary Figure S5). Three clones from the same cultivated accession were used as biological replicates.

The three floral developmental stages collected correspond to stages 1, 3, and 4 from De Keyser et al. [93]: early floral bud (closed bud, enclosed in inflorescence scales), late floral bud (“candle” stage, inflorescence scales abscised), and open flower, respectively. Leaves, late floral buds, and open flowers were immediately flash-frozen in liquid nitrogen and then stored at −70 °C. We removed the indumentum from leaves by gentle rubbing prior to freezing. We collected inflorescence buds on ice, then dissected individual flower buds, flash-froze them, and stored them at −70 °C. Early floral buds were 7–13 mm long for *R. beyerinckianum* and 4–8 mm for *R. taxifolium.* Late floral buds were 22–28 mm long for *R. beyerinckianum* and 11–17 mm for *R. taxifolium*. Open flowers were 33–37 mm long for *R. beyerinckianum* and 19–25 mm for *R. taxifolium*.

Preliminary analyses showed that flower symmetry candidate genes had the highest expression in late floral buds and open flowers. Therefore, we selected late floral buds for examining gene expression across floral organs. Late floral buds were collected on ice and then carefully dissected into a single dorsal petal, two lateral petals, two ventral petals, five upper stamens (including dorsal and lateral stamens), five lower stamens (including ventral stamens), and a pistil, which was dissected from the flower just above the nectary disc. Floral organs were frozen in liquid nitrogen and stored at −70 °C.

### 4.5. RNA Extraction and cDNA Synthesis

Total RNA was extracted from early floral buds, late floral buds, open flowers, and leaves using the Spectrum^™^ Plant Total RNA Kit (Sigma-Aldrich, St. Louis, MO, USA). Four samples from each floral developmental stage, or leaves, were pooled and homogenized in chilled mortars and pestles and immediately extracted for RNA or stored at −70 °C.

For dissected floral organs, organs from four (*R. beyerinckianum*) or 12 (*R. taxifolium)* late floral buds were pooled and homogenized. Total RNA was extracted using the Spectrum^™^ Plant Total RNA Kit, according to the manufacturer’s protocol with the following modifications. Tubes were vortexed for 1 min after addition of the Lysis Solution, and 750 µL of Binding Solution was used in the binding step. For difficult tissues with low yield, extraction was repeated using </= 50 mg of tissue, 1 mL of Lysis Solution, and 750 µL per 0.5 mL of Binding Solution in the binding step. For very low yields, multiple preps of the same tissue were performed, and then the RNA was eluted sequentially with the same elution buffer. In a few cases, extracts were cleaned and concentrated using Agencourt^®^ RNAClean^®^ XP (Beckman Coulter, Indianapolis, IN, USA) as needed. RNA quantity was estimated using a Qubit^®^ 2.0 Fluorometer (Invitrogen^™^, Life Technologies^™^, Carlsbad, CA, USA).

One µg of total RNA was used for cDNA synthesis with the SuperScript^™^ III First-Strand Synthesis System (Invitrogen^™^, Life Technologies^™^, Carlsbad, CA, USA). First, the RNA was treated with DNAse I (New England Biolabs^®^ Inc., Ipswich, MA, USA) and then used as template for cDNA synthesis, according to the manufacturer’s protocols. The cDNA was checked for genomic contamination by performing RT-PCR with *ELONGATION FACTOR 1-ALPHA* (*EF1A*) primers that spanned introns (Supplementary Table S3).

### 4.6. 3′RACE of Rhododendron CYC Orthologs

In order to obtain 3′ untranslated region (UTR) sequences for locus-specific primer designs for RT-PCR of *Rhododendron CYC* orthologs, we used a modified protocol of 3′ RACE [94,95] on *R. beyerinckianum* and *R. taxifolium.* We pooled cDNA from the developmental stages above and used 1 µL of 1:2 cDNA as template in PCR with Phusion^®^ High-Fidelity DNA Polymerase and ReadyMade^TM^ Anchored Oligo dT (20) (Integrated DNA Technologies, Coralville, IA, USA) and Rbey_CYC2_3RACE_F1 primers (Supplementary Table S3). PCR conditions were an initial denaturation at 98 °C for 30 s, followed by five cycles of denaturation at 98 °C for 10 s, annealing at 45 °C for 30s, and extension at 72 °C for 3 min, followed by 25 cycles of denaturation at 98 °C for 10 s, annealing at 55 °C for 30s, and extension at 72 °C for 3 min, and a final extension at 72 °C for 10 min. We only obtained 3′ RACE products from *R. beyerinckianum*; this was diluted 1:20 in Tris-EDTA for reamplification with the nested primer Rbey_CYC2_3RACE_F2 (Supplementary Table S3) and the ReadyMade^TM^ Anchored Oligo dT (20) primer using the same PCR conditions as the first round above. The second round of 3′ RACE was gel-excised and cloned as described above, using A-tailing with Taq, ligation into the pCR^™^2.1-TOPO^®^ cloning vector, and 100 positive clones sequenced by GENEWIZ with M13 primers and an internal sequencing primer (Supplementary Table S3). We also designed a paralog-specific 3′ RACE nested primer for *RhbCYC2.5* (Rbey_CYC2.5_3RACE_F2, Supplementary Table S3) as the nested primer above did not capture the 3′ UTR from this paralog. We performed reamplification as above, sequencing nine positive clones.

Due to the presence of alternative transcripts in the 3′ UTRs of *RhbCYC2.1, RhbCYC2.2, RhbCYC2.4,* and *RhbCYC2.5*, we designed primers to amplify these regions from gDNA (Supplementary Table S3). We also attempted to use these primers to amplify 3′ UTRs from gDNA of *R. taxifolium* but only obtained 3′ UTRs for *RhtCYC2.4*. 3′ UTR PCR conditions were an initial denaturation at 98 °C for 30 s, followed by 35 cycles of denaturation at 98 °C for 10 s, annealing at 58–68 °C for 30 s (Supplementary Table S3), and extension at 72 °C for 90 s, with a final extension at 72 °C for 10 min. PCR products were gel-extracted or cleaned, then cloned using the TOPO^TM^ XL-2 Complete PCR Cloning Kit (Invitrogen^™^, Life Technologies^™^, Carlsbad, CA, USA), according to the manufacturer’s directions. Ten positive clones were sequenced by GENEWIZ with their M13 or T3/T7 primers and internal sequencing primers (Supplementary Table S3). Nucleotide sequences were assembled in Sequencher and aligned in Mesquite.

### 4.7. Expression Analyses of Rhododendron CYC, RAD, and DIV Orthologs

Paralog-specific RT-PCR primers were designed for *CYC*, *DIV*, and *RAD* orthologs in *R. beyerinckianum* and *R. taxifolium* from sequences obtained above. After trying several reference genes previously tested in *Rhododendron* [96,97], we chose *ACTIN5* (*ACT5*). We used *R. molle* G.Don *ACT5* [97] to search the *R. delavayi* and *R. williamsianum* genomes and *R. beyerinckianum* and *R. taxifolium* transcriptomes with BLAST+ to obtain additional sequences. We designed RT-PCR primers using Primer3web v4.1.0 [98] or manually, and verified suitability using the OligoAnalyzer Tool (Supplementary Table S3). Primer pairs were constructed to generate RT-PCR products 206–550 bp. To verify that primers isolated individual paralogs of each gene, we amplified from both gDNA and cDNA, cloned, and confirmed via sequencing.

To examine the expression of flower symmetry candidate genes in *R. beyerinckianum* and *R. taxifolium,* RT-PCR was performed in a 25-µL reaction containing 1X reaction buffer, 0.2 mM dNTPs, 5 pmol of each primer, and 1.25 U of Phusion^®^ High-Fidelity DNA Polymerase. cDNA was used at dilutions of 1:0–1:4 (as determined by *ACT5* expression levels for each replicate). Reactions were processed in a T100^™^ Thermal Cycler with an initial denaturation at 98 °C for 20 s, followed by 30 cycles of denaturation at 98 °C for 10 s, annealing at 56–68 °C for 30 s, and extension at 72 °C for 30 s, and a final extension at 72 °C for 5 min (Supplementary Table S3). Loading volumes for each floral stage and dissected organ were determined by quantification of gel images using the Gel Doc^™^ EZ Imaging System and Image Lab v5.2.1 (Bio-Rad Laboratories, Inc., Hercules, CA, USA). Negative and positive controls were run alongside experimental samples. Loading volumes 2–24 µL were run on a 1.2% TAE agarose gel containing a 1:10,000 dilution of GelRed^®^ Nucleic Acid Gel Stain (Biotium, Inc., Hayward, CA, USA) for 90 min at 7.5 V/cm. Gels were visualized and photographed using the Gel Doc^™^ EZ Imaging System with auto-exposure settings. We reported gene expression from RT-PCR if the pattern was observed in at least two replicates. We also quantified gene expression from RT-PCR results using the Gel Doc^™^ EZ Imaging System and Image Lab and averaged this across the three biological replicates to summarize expression patterns for each gene in each species.

## 5. Conclusions

*Rhododendron*, an early diverging lineage of asterids, exhibits conserved expression patterns of *CYC* orthologs in bilaterally symmetric flowers as other asterids. In the derived, tropical sect. *Schistanthe*, we found extensive tandem duplication in *CYC* orthologs and restriction of gene expression to the dorsal and lateral petals and upper stamens in bilaterally symmetric *R. beyerinckianum*. These *CYC* orthologs are associated with differential petal, stamen, and pistil growth in this species, potentially contributing to its adaxially curved and positioned floral organs. We also found a potentially novel role for *CYC* orthologs in style elongation and curvature. In a radially symmetric species from the sister clade, *R. taxifolium*, ubiquitous expression of two *CYC* orthologs, loss of one *CYC* ortholog, and reduced expression in another ortholog act in concert to produce shorter and equal petals, stamens, and styles. In contrast, we found ubiquitous expression of *RAD* and *DIV* orthologs in both species, as well as a difference in gene structure in a *RAD* ortholog, which suggests that *RAD* may not be regulated by *CYC* orthologs. *CYC* orthologs are likely the main regulators of floral symmetry in *Rhododendron*, potentially promoting growth in the absence of interactions with *RAD* orthologs. Our study provides a glimpse of the potential variation in *CYC* orthologs in *Rhododendron* that likely contributed to the extensive diversity in floral symmetry in this genus.

## Data Availability

The data presented in this study are openly available in TreeBASE for alignments and phylogenies (http://purl.org/phylo/treebase/phylows/study/TB2:S28224) and GenBank for sequences generated in this study (Supplementary Table S2).

## Supplementary Materials

The following are available online at https://www.mdpi.com/article/10.3390/plants10101994/s1, Figure S1: Gene structure of Rhododendron beyerinckianum CYCLOIDEA (CYC), RADIALIS (RAD), and DIVARICATA (DIV) orthologs; Figure S2: Protein alignment of CYCLOIDEA (CYC) orthologs from Rhododendron sect. Schistanthe; Figure S3: Protein alignment of RADIALIS (RAD) orthologs from Rhododendron sect. Schistanthe; Figure S4: Protein alignment of DIVARICATA (DIV) orthologs from Rhododendron sect. Schistanthe; Figure S5: Gene expression of CYCLOIDEA (CYC), RADIALIS (RAD), and DIVARICATA (DIV) orthologs across floral developmental stages in two Rhododendron species with different floral symmetries; Figure S6: Gene expression of CYCLOIDEA (CYC), RADIALIS (RAD), and DIVARICATA (DIV) orthologs across floral organs in two Rhododendron species with different floral symmetries; Table S1: Rhododendron samples and outgroups collected for this study; Table S2: Sources of DNA sequences used in this study; Table S3: Primer sequences and PCR conditions used in this study.

## References

[1] Neal P.R., Dafni A., Giurfa M. (1998). Floral symmetry and its role in plant-pollinator systems: Terminology, distribution, and hypotheses. Annu. Rev. Ecol. Syst..

[2] Sargent R.D. (2004). Floral symmetry affects speciation rates in angiosperms. Proc. R. Soc. B Boil Sci..

[3] O’Meara B.C., Smith S.D., Armbruster W.S., Harder L., Hardy C.R., Hileman L., Hufford L., Litt A., Magallón S., Smith S. (2016). Non-equilibrium dynamics and floral trait interactions shape extant angiosperm diversity. Proc. R. Soc. B Boil Sci..

[4] Citerne H., Jabbour F., Nadot S., Damerval C. (2010). The evolution of floral symmetry. Adv. Bot. Res..

[5] Reyes E., Sauquet H., Nadot S. (2016). Perianth symmetry changed at least 199 times in angiosperm evolution. Taxon.

[6] Luo D., Carpenter R., Vincent C., Copsey L., Coen E. (1996). Origin of floral asymmetry in Antirrhinum. Nat. Cell Biol..

[7] Cubas P., Lauter N., Doebley J., Coen E. (1999). The TCP domain: A motif found in proteins regulating plant growth and development. Plant J..

[8] Luo D., Carpenter R., Copsey L., Vincent C., Clark J., Coen E. (1999). Control of organ asymmetry in flowers of antirrhinum. Cell.

[9] Gübitz T., Caldwell A., Hudson A. (2003). Rapid molecular evolution of *Cycloidea*-like genes in *Antirrhinum* and its relatives. Mol. Biol. Evol..

[10] Hileman L.C., Baum D.A. (2003). Why do paralogs persist? Molecular evolution of *Cycloidea* and related floral symmetry genes in *Antirrhineae* (*Veronicaceae*). Mol. Biol. Evol..

[11] Li M., Zhang D., Gao Q., Luo Y., Zhang H., Ma B., Chen C., Whibley A., Zhang Y., Cao Y. (2019). Genome structure and evolution of *Antirrhinum majus* L.. Nat. Plants.

[12] Galego L. (2002). Role of DIVARICATA in the control of dorsoventral asymmetry in *Antirrhinum* flowers. Genes Dev..

[13] Corley S.B., Carpenter R., Copsey L., Coen E. (2005). Floral asymmetry involves an interplay between TCP and MYB transcription factors in *Antirrhinum*. Proc. Natl. Acad. Sci. USA.

[14] Costa M.M., Fox S., Hanna A.I., Baxter C., Coen E. (2005). Evolution of regulatory interactions controlling floral asymmetry. Development.

[15] Raimundo J., Sobral R., Bailey P., Azevedo H., Galego L., Almeida J., Coen E., Costa M.M.R. (2013). A subcellular tug of war involving three MYB-like proteins underlies a molecular antagonism in *Antirrhinum* flower asymmetry. Plant J..

[16] Navaud O., Dabos P., Carnus E., Tremousaygue D., Hervé C. (2007). TCP Transcription factors predate the emergence of land plants. J. Mol. Evol..

[17] Howarth D.G., Donoghue M.J. (2005). Duplications in CYC-like genes from dipsacales correlate with floral form. Int. J. Plant Sci..

[18] Howarth D.G., Donoghue M.J. (2006). Phylogenetic analysis of the “ECE” (CYC/TB1) clade reveals duplications predating the core eudicots. Proc. Natl. Acad. Sci. USA.

[19] Madrigal Y., Alzate J.F., Pabón-Mora N. (2017). Evolution and expression patterns of TCP genes in asparagales. Front. Plant Sci..

[20] Busch A., Zachgo S. (2009). Flower symmetry evolution: Towards understanding the abominable mystery of angiosperm radiation. Bioessays.

[21] Specht C., Howarth D.G. (2015). Adaptation in flower form: A comparative evodevo approach. New Phytol..

[22] Jabbour F., Cossard G., Le Guilloux M., Sannier J., Nadot S., Damerval C. (2014). Specific duplication and dorsoventrally asymmetric expression patterns of cycloidea-like genes in zygomorphic species of *Ranunculaceae*. PLoS ONE.

[23] Hileman L.C. (2014). Trends in flower symmetry evolution revealed through phylogenetic and developmental genetic advances. Philos. Trans. R. Soc. B Biol. Sci..

[24] Yanhui C., Xiaoyuan Y., Kun H., Meihua L., Jigang L., Zhaofeng G., Zhiqiang L., Yunfei Z., Xiaoxiao W., Xiaoming Q. (2006). The MYB transcription factor superfamily of arabidopsis: Expression analysis and phylogenetic comparison with the rice MYB family. Plant Mol. Biol..

[25] Raimundo J., Sobral R., Laranjeira S., Costa M.M.R. (2018). Successive domain rearrangements underlie the evolution of a regulatory module controlled by a small interfering peptide. Mol. Biol. Evol..

[26] Howarth D.G., Donoghue M.J. (2009). Duplications and expression of divaricata-like genes in dipsacales. Mol. Biol. Evol..

[27] Boyden G.S., Donoghue M.J., Howarth D.G. (2012). Duplications and expression of radialis-like genes in dipsacales. Int. J. Plant Sci..

[28] Madrigal Y., Alzate J.F., González F., Pabón-Mora N. (2019). Evolution of radialis and divaricata gene lineages in flowering plants with an expanded sampling in non-core eudicots. Am. J. Bot..

[29] Gao A., Zhang J., Zhang W. (2017). Evolution of rad- and div-like genes in plants. Int. J. Mol. Sci..

[30] Zhou X.-R., Wang Y.-Z., Smith J.F., Chen R. (2008). Altered expression patterns of TCP and MYB genes relating to the floral developmental transition from initial zygomorphy to actinomorphy in *Bournea* (*Gesneriaceae*). New Phytol..

[31] Preston J.C., Kost M.A., Hileman L.C. (2009). Conservation and diversification of the symmetry developmental program among close relatives of snapdragon with divergent floral morphologies. New Phytol..

[32] Zhong J., Preston J.C., Hileman L.C., Kellogg E.A. (2017). Repeated and diverse losses of corolla bilateral symmetry in the *Lamiaceae*. Ann. Bot..

[33] Preston J.C., Martinez C.C., Hileman L. (2011). Gradual disintegration of the floral symmetry gene network is implicated in the evolution of a wind-pollination syndrome. Proc. Natl. Acad. Sci. USA.

[34] Reardon W., Gallagher P., Nolan K.M., Wright H., Rubio M.D.L.C.C., Bragalini C., Lee C., Fitzpatrick D., Corcoran K., Wolff K. (2014). Different outcomes for the MYB floral symmetry genes divaricata and radialis during the evolution of derived actinomorphy in *Plantago*. New Phytol..

[35] Hsin K.-T., Wang C.-N. (2018). Expression shifts of floral symmetry genes correlate to flower actinomorphy in East Asia endemic Conandron ramondioides (*Gesneriaceae*). Bot. Stud..

[36] Liu J., Wu J., Yang X., Wang Y. (2020). Regulatory pathways of CYC -like genes in patterning floral zygomorphy exemplified in *Chirita pumila*. J. Syst. Evol..

[37] Zhong J., Kellogg E.A. (2015). Stepwise evolution of corolla symmetry in cycloidea2 -like and radialis -like gene expression patterns in *Lamiales*. Am. J. Bot..

[38] Baxter C.E.L., Costa M.M., Coen E.S. (2007). Diversification and co-option of RAD-like genes in the evolution of floral asymmetry. Plant J..

[39] Zhang F., Liu X., Zuo K., Zhang J., Sun X., Tang K. (2011). Molecular cloning and characterization of a novel *Gossypium barbadense* L. RAD-like gene. Plant Mol. Biol. Rep..

[40] Zhang F., Liu X., Zuo K., Sun X., Tang K. (2011). Molecular cloning and expression analysis of a novel SANT/MYB gene from *Gossypium barbadense*. Mol. Biol. Rep..

[41] Yang B., Song Z., Li C., Jiang J., Zhou Y., Wang R., Wang Q., Ni C., Liang Q., Chen H. (2018). RSM1, an arabidopsis MYB protein, interacts with HY5/HYH to modulate seed germination and seedling development in response to abscisic acid and salinity. PLoS Genet..

[42] Stevens P.F., Luteyn J., Oliver E.G.H., Bell T.L., Brown E.A., Crowden R.K., George A.S., Jordan G.J., Ladd P., Lemson K. (2004). Ericaceae. The Families and Genera of Vascular Plants: Flowering Plants, Dicotyledons, Celastrales, Oxalidales, Rosales, Cornales, Ericales.

[43] Byng J.W., Chase M.W., Christenhusz M.J.M., Fay M.F., Judd W.S., Mabberley D.J., Sennikov A.N., Soltis D.E., Soltis P.S., Stevens P.F. (2016). An update of the angiosperm phylogeny group classification for the orders and families of flowering plants: APG IV. Bot. J. Linn. Soc..

[44] Goetsch L., Eckert A.J., Hall B.D. (2005). The molecular systematics of *Rhododendron* (*Ericaceae*): A phylogeny based upon RPB2 gene sequences. Syst. Bot..

[45] Shrestha N., Wang Z., Su X., Xu X., Lyu L., Liu Y., Dimitrov D., Kennedy J.D., Wang Q., Tang Z. (2018). Global patterns of *Rhododendron* diversity: The role of evolutionary time and diversification rates. Glob. Ecol. Biogeogr..

[46] Berry E., Sharma S.K., Pandit M.K., Geeta R. (2017). Evolutionary correlation between floral monosymmetry and corolla pigmentation patterns in *Rhododendron*. Plant Syst. Evol..

[47] Stevens P.F. (1976). The altitudinal and geographical distributions of flower types in *Rhododendron* section *Vireya*, especially in the *Papuasian* species, and their significance. Bot. J. Linn. Soc..

[48] Craven L., Dăneţ F., Veldkamp J., Goetsch L., Hall B. (2011). Vireya *Rhododendrons*: Their monophyly and classification (*Ericaceae*, *Rhododendron* section *Schistanthe*). Blumea Biodivers. Evol. Biogeogr. Plants.

[49] Zhang L., Xu P., Cai Y., Ma L., Li S., Li S., Xie W., Song J., Peng L., Yan H. (2017). The draft genome assembly of *Rhododendron delavayi* Franch. var. delavayi. GigaScience.

[50] Colle M., Leisner C.P., Wai C.M., Ou S., Bird K.A., Wang J., Wisecaver J.H., Yocca A.E., Alger E.I., Tang H. (2019). Haplotype-phased genome and evolution of phytonutrient pathways of tetraploid blueberry. GigaScience.

[51] Soza V.L., Lindsley D., Waalkes A., Ramage E., Patwardhan R.P., Burton J.N., Adey A., Kumar A., Qiu R., Shendure J. (2019). The *Rhododendron* genome and chromosomal organization provide insight into shared whole-genome duplications across the heath family (*Ericaceae*). Genome Biol. Evol..

[52] Wu H., Ma T., Kang M., Ai F., Zhang J., Dong G., Liu J. (2019). A high-quality *Actinidia chinensis* (kiwifruit) genome. Hortic. Res..

[53] Yang F.-S., Nie S., Liu H., Shi T.-L., Tian X.-C., Zhou S.-S., Bao Y.-T., Jia K.-H., Guo J.-F., Zhao W. (2020). Chromosome-level genome assembly of a parent species of widely cultivated azaleas. Nat. Commun..

[54] Ramage E., Soza V.L., Hall B.D. (2021). Transcriptome Assemblies across the Genus Rhododendron (Ericaceae).

[55] Soza V.L., Kriebel R., Ramage E., Hall B.D., Twyford A.D. (2021). The Symmetry Spectrum in a Hybridizing, Tropical Group of Rhododendrons.

[56] Gillespie E., Kron K. (2010). Molecular phylogenetic relationships and a revised classification of the subfamily *Ericoideae* (*Ericaceae*). Mol. Phylogenetics Evol..

[57] Rose J.P., Kleist T.J., Löfstrand S.D., Drew B.T., Schönenberger J., Sytsma K.J. (2018). Phylogeny, historical biogeography, and diversification of angiosperm order Ericales suggest ancient Neotropical and East Asian connections. Mol. Phylogenetics Evol..

[58] Chapman M.A., Leebens-Mack J.H., Burke J.M. (2008). Positive selection and expression divergence following gene duplication in the sunflower *Cycloidea* gene family. Mol. Biol. Evol..

[59] Gaut B.S., Wright S.I., Rizzon C., Dvorak J., Anderson L.K. (2007). Recombination: An underappreciated factor in the evolution of plant genomes. Nat. Rev. Genet..

[60] Gao Q., Tao J.-H., Yan D., Wang Y.-Z., Li Z.-Y. (2008). Expression differentiation of CYC-like floral symmetry genes correlated with their protein sequence divergence in *Chirita heterotricha* (*Gesneriaceae*). Dev. Genes Evol..

[61] Song C.-F., Lin Q.-B., Liang R.-H., Wang Y.-Z. (2009). Expressions of ECE-CYC2 clade genes relating to abortion of both dorsal and ventral stamens in Opithandra (*Gesneriaceae*). BMC Evol. Biol..

[62] Howarth D.G., Martins T., Chimney E., Donoghue M.J. (2011). Diversification of *Cycloidea* expression in the evolution of bilateral flower symmetry in *Caprifoliaceae* and *Lonicera* (*Dipsacales*). Ann. Bot..

[63] Yang X., Pang H.-B., Liu B.-L., Qiu Z.-J., Gao Q., Wei L., Dong Y., Wang Y.-Z. (2012). Evolution of double positive autoregulatory feedback loops in *Cycloidea* 2 clade genes is associated with the origin of floral zygomorphy. Plant Cell.

[64] Zhong J., Kellogg E. (2015). Duplication and expression of CYC2-like genes in the origin and maintenance of corolla zygomorphy in Lamiales. New Phytol..

[65] Hsin K.-T., Lu J.-Y., Möller M., Wang C.-N. (2019). Gene duplication and relaxation from selective constraints of GCYC genes correlated with various floral symmetry patterns in Asiatic *Gesneriaceae tribe Trichosporeae*. PLoS ONE.

[66] Garcês H.M.P., Spencer V.M., Kim M. (2016). Control of floret symmetry by RAY3, SvDIV1B, and SvRAD in the capitulum of *Senecio vulgaris*. Plant Physiol..

[67] Hsu H.-C., Wang C.-N., Liang C.-H., Wang C.-C., Kuo Y.-F. (2017). Association between petal form variation and CYC2-like genotype in a hybrid line of *Sinningia speciosa*. Front. Plant Sci..

[68] Broholm S.K., Tähtiharju S., Laitinen R.A.E., Albert V.A., Teeri T., Elomaa P. (2008). A TCP domain transcription factor controls flower type specification along the radial axis of the Gerbera (*Asteraceae*) inflorescence. Proc. Natl. Acad. Sci. USA.

[69] Reardon W., Fitzpatrick D., Fares M.A., Nugent J.M. (2009). Evolution of flower shape in *Plantago lanceolata*. Plant Mol. Biol..

[70] Pang H.-B., Sun Q.-W., He S.-Z., Wang Y.-Z. (2010). Expression pattern of CYC-like genes relating to a dorsalized actinomorphic flower in Tengia (*Gesneriaceae*). J. Syst. Evol..

[71] Larson D.A., Walker J.F., Vargas O.M., Smith S.A. (2020). A consensus phylogenomic approach highlights paleopolyploid and rapid radiation in the history of Ericales. Am. J. Bot..

[72] Altschul S.F., Madden T.L., Schäffer A.A., Zhang J., Zhang Z., Miller W., Lipman D.J. (1997). Gapped BLAST and PSI-BLAST: A new generation of protein database search programs. Nucleic Acids Res..

[73] Camacho C., Coulouris G., Avagyan V., Ma N., Papadopoulos J.S., Bealer K., Madden T.L. (2009). BLAST+: Architecture and applications. BMC Bioinform..

[74] Waterhouse A.M., Procter J.B., Martin D.M.A., Clamp M., Barton G.J. (2009). Jalview version 2—a multiple sequence alignment editor and analysis workbench. Bioinformatics.

[75] Maddison W.P., Maddison D.R. Mesquite: A Modular System for Evolutionary Analysis. Version 3.70. http://www.mesquiteproject.org.

[76] Martin D.P., Murrell B., Golden M., Khoosal A., Muhire B. (2015). RDP4: Detection and analysis of recombination patterns in virus genomes. Virus Evol..

[77] Martin D., Rybicki E. (2000). RDP: Detection of recombination amongst aligned sequences. Bioinformatics.

[78] Padidam M., Sawyer S., Fauquet C.M. (1999). Possible emergence of new geminiviruses by frequent recombination. Virology.

[79] Smith J.M. (1992). Analyzing the mosaic structure of genes. J. Mol. Evol..

[80] Posada D., Crandall K.A. (2001). Evaluation of methods for detecting recombination from DNA sequences: Computer simulations. Proc. Natl. Acad. Sci. USA.

[81] Salminen M., Carr J.K., Burke D.S., McCutchan F.E. (1995). Identification of breakpoints in intergenotypic recombinants of HIV type 1 by bootscanning. AIDS Res. Hum. Retrovir..

[82] Martin D., Posada D., Crandall K., Williamson C. (2005). A Modified bootscan algorithm for automated identification of recombinant sequences and recombination breakpoints. AIDS Res. Hum. Retrovir..

[83] Gibbs M.J., Armstrong J.S., Gibbs A.J. (2000). Sister-scanning: A Monte Carlo procedure for assessing signals in recombinant sequences. Bioinformatics.

[84] Citerne H.L., Le Guilloux M., Sannier J., Nadot S., Damerval C. (2013). Combining phylogenetic and syntenic analyses for understanding the evolution of TCP ECE genes in eudicots. PLoS ONE.

[85] Darriba D., Taboada G.L., Doallo R., Posada D. (2012). jModelTest 2: More models, new heuristics and parallel computing. Nat. Methods.

[86] Guindon S., Gascuel O. (2003). A simple, fast, and accurate algorithm to estimate large phylogenies by maximum likelihood. Syst. Biol..

[87] Miller M.A., Pfeiffer W., Schwartz T. Creating the CIPRES science gateway for inference of large phylogenetic trees. Proceedings of the 2010 Gateway Computing Environments Workshop (GCE).

[88] Akaike H. (1974). A new look at the statistical model identification. Selected Papers of Hirotugu Akaike.

[89] Huelsenbeck J.P., Ronquist F. (2001). Mrbayes: Bayesian inference of phylogenetic trees. Bioinformatics.

[90] Ronquist F., Huelsenbeck J.P. (2003). MrBayes 3: Bayesian phylogenetic inference under mixed models. Bioinformatics.

[91] Yang Z., Rannala B. (1997). Bayesian phylogenetic inference using DNA sequences: A markov chain Monte Carlo method. Mol. Biol. Evol..

[92] Rambaut A. (2016). FigTree Version 1.4.3. http://tree.bio.ed.ac.uk/software/figtree/.

[93] De Keyser E., Desmet L., Van Bockstaele E., De Riek J. (2013). How to perform RT-qPCR accurately in plant species? A case study on flower colour gene expression in an azalea (*Rhododendron simsii* hybrids) mapping population. BMC Mol. Biol..

[94] Loh E. (1991). Anchored PCR: Amplification with single-sided specificity. Methods.

[95] Scotto–Lavino E., Du G., Frohman M.A. (2006). 3′ End cDNA amplification using classic RACE. Nat. Protoc..

[96] Yi S., Qian Y., Han L., Sun Z., Fan C., Liu J., Ju G. (2012). Selection of reliable reference genes for gene expression studies in *Rhododendron micranthum* Turcz. Sci. Hortic..

[97] Xiao Z., Sun X., Liu X., Li C., He L., Chen S., Su J. (2016). Selection of reliable reference genes for gene expression studies on *Rhododendron molle* G. Don. Front. Plant Sci..

[98] Untergasser A., Cutcutache I., Koressaar T., Ye J., Faircloth B.C., Remm M., Rozen S.G. (2012). Primer 3—new capabilities and interfaces. Nucleic Acids Res..

