# Supplementary material for: Gene Duplication and Differential Expression of Flower Symmetry Genes in Rhododendron (Ericaceae)
Source: Plants (Basel). 2021 Sep 23;10(10):1994. doi: 10.3390/plants10101994 (PMC8541606; doi:10.3390/plants10101994)
Supplement: Supplementary file 1 [file plants-10-01994-s001.zip › FigS1.Rhb.gene.structure.060821.pdf]

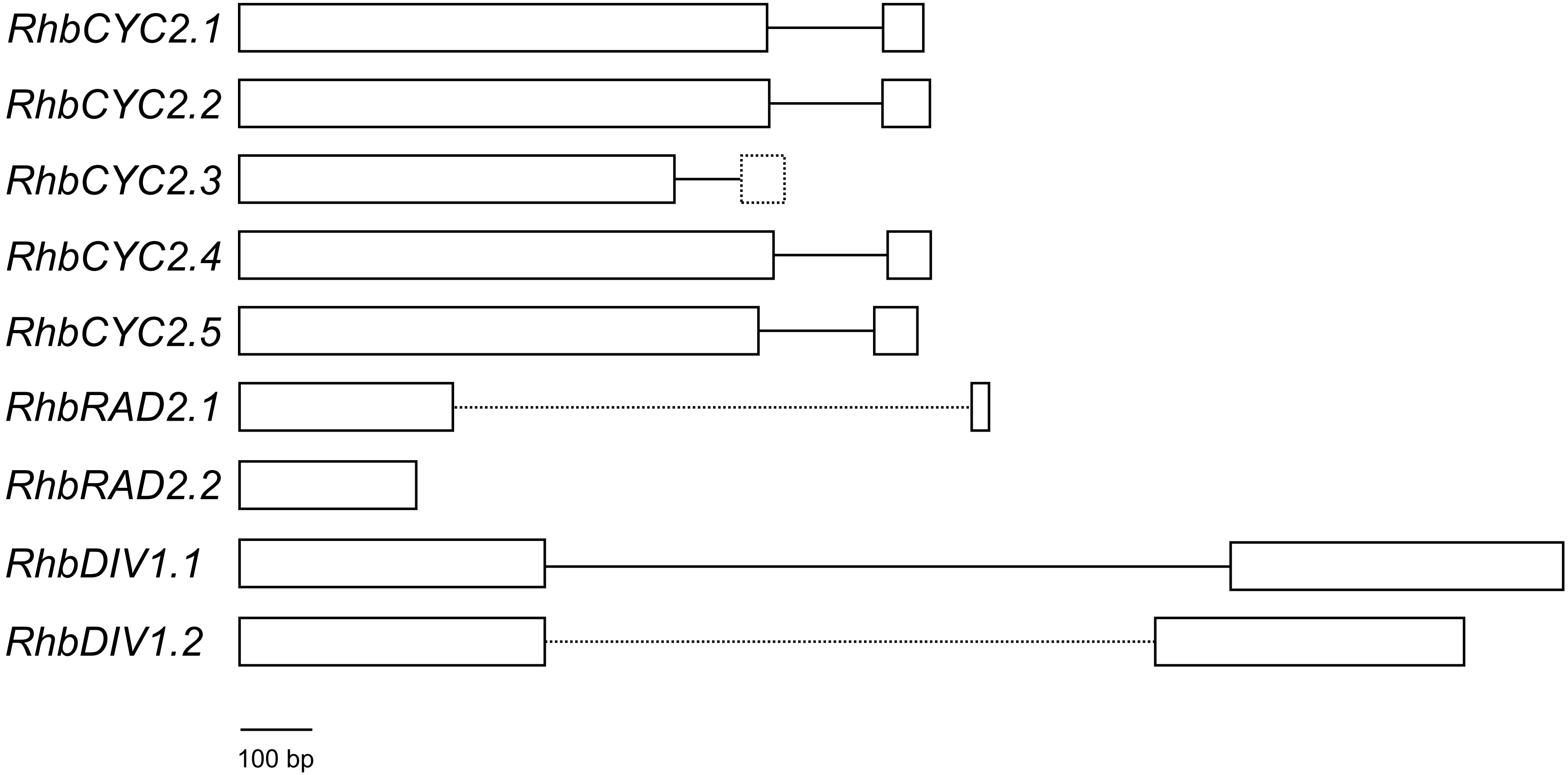

Supplementary Figure S1. **Gene structure of *Rhododendron beyerinckianum CYCLOIDEA* (*CYC*), *RADIALIS* (*RAD*), and *DIVARICATA* (*DIV*) orthologs.**
Exons denoted by rectangles; introns denoted by lines. Dotted lines indicate approximate length.
