# Supplementary material for: Gene Duplication and Differential Expression of Flower Symmetry Genes in Rhododendron (Ericaceae)
Source: Plants (Basel). 2021 Sep 23;10(10):1994. doi: 10.3390/plants10101994 (PMC8541606; doi:10.3390/plants10101994)
Supplement: Supplementary file 1 [file plants-10-01994-s001.zip › FigS3.RhRAD2proteins.060821.pdf]

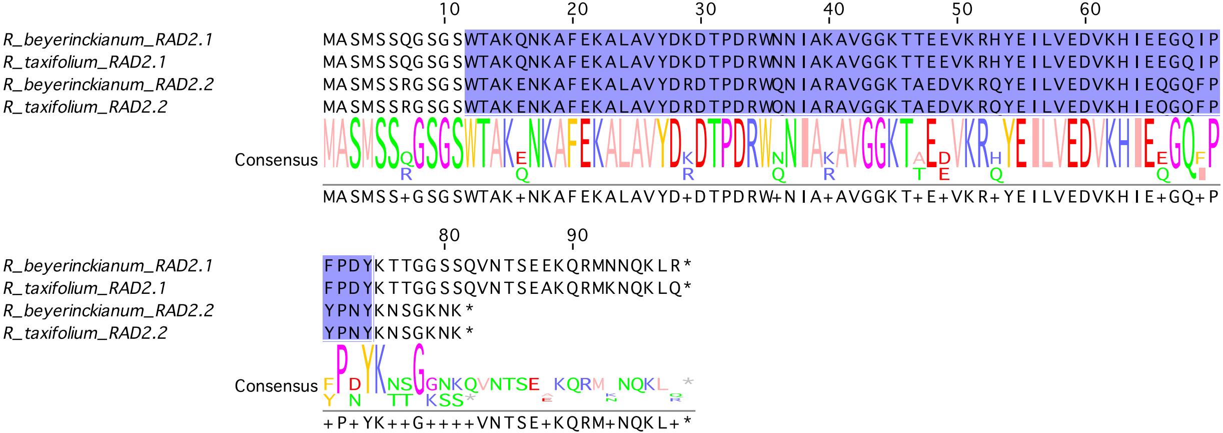

Supplementary Figure S3. Protein alignment of *RADIALIS* (*RAD*) orthologs from *Rhododendron* sect. *Schistanthe*.
MYBI domain highlighted in blue.
