# Supplementary material for: Gene Duplication and Differential Expression of Flower Symmetry Genes in Rhododendron (Ericaceae)
Source: Plants (Basel). 2021 Sep 23;10(10):1994. doi: 10.3390/plants10101994 (PMC8541606; doi:10.3390/plants10101994)
Supplement: Supplementary file 1 [file plants-10-01994-s001.zip › FigS5.RTPCR_stages_reps.060821.pdf]

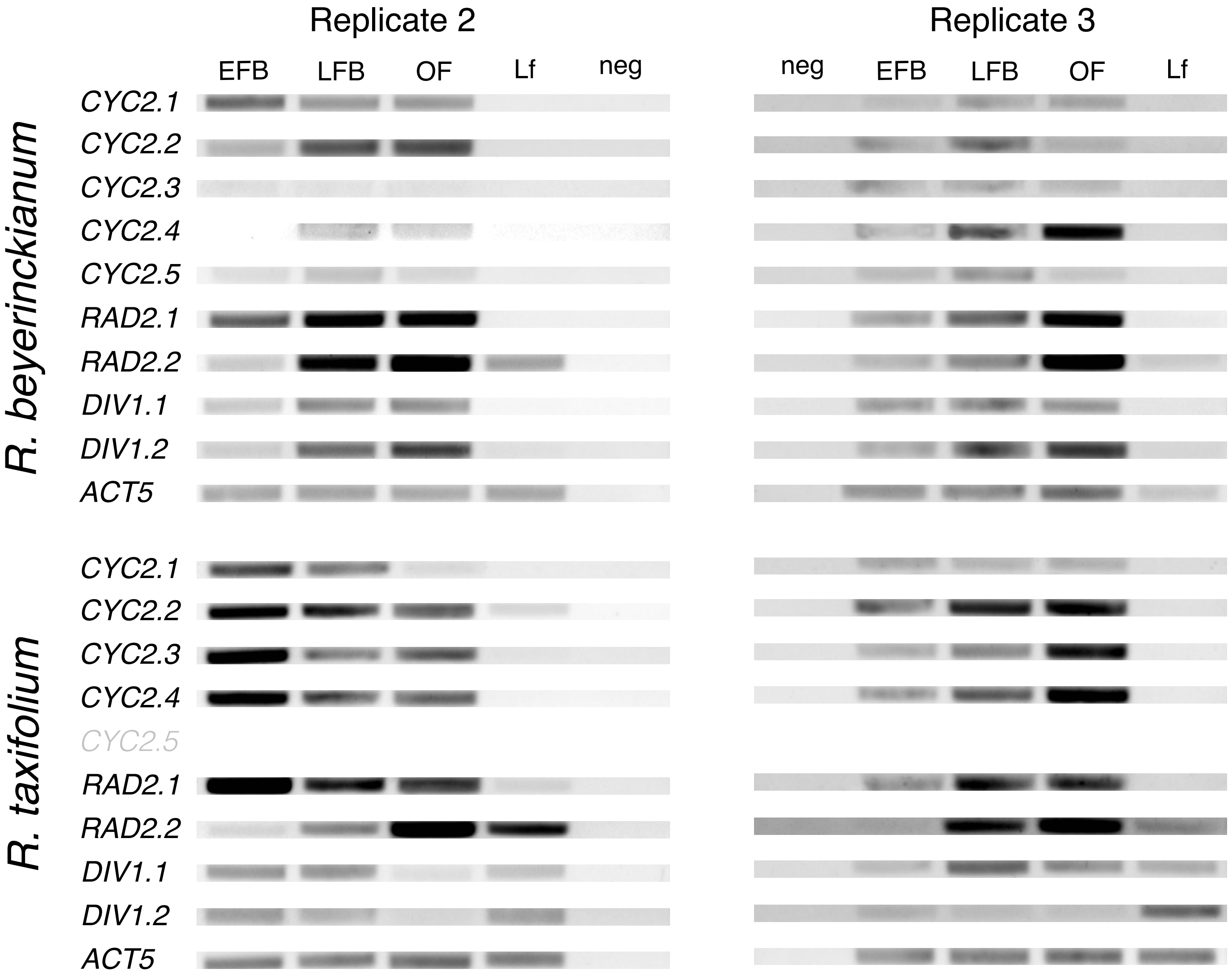

Supplementary Figure S5. Gene expression of *CYCLOIDEA* (*CYC*), *RADIALIS* (*RAD*), and *DIVARICATA* (*DIV*) orthologs across floral developmental stages in two *Rhododendron* species with different floral symmetries. Gene expression shown by RT-PCR for two additional replicates. Floral developmental stages sampled are early floral bud (EFB), late floral bud (LFB), and open flower (OF). Vegetative (Lf) and negative (neg) controls also shown. *ACTIN5* (*ACT5*) used as a reference gene. *R. beyerinckianum* is bilaterally symmetric; *R. taxifolium* is radially symmetric.
