# Supplementary material for: Gene Duplication and Differential Expression of Flower Symmetry Genes in Rhododendron (Ericaceae)
Source: Plants (Basel). 2021 Sep 23;10(10):1994. doi: 10.3390/plants10101994 (PMC8541606; doi:10.3390/plants10101994)
Supplement: Supplementary file 1 [file plants-10-01994-s001.zip › FigS6.RTPCR_cs_organs_reps.060821.pdf]

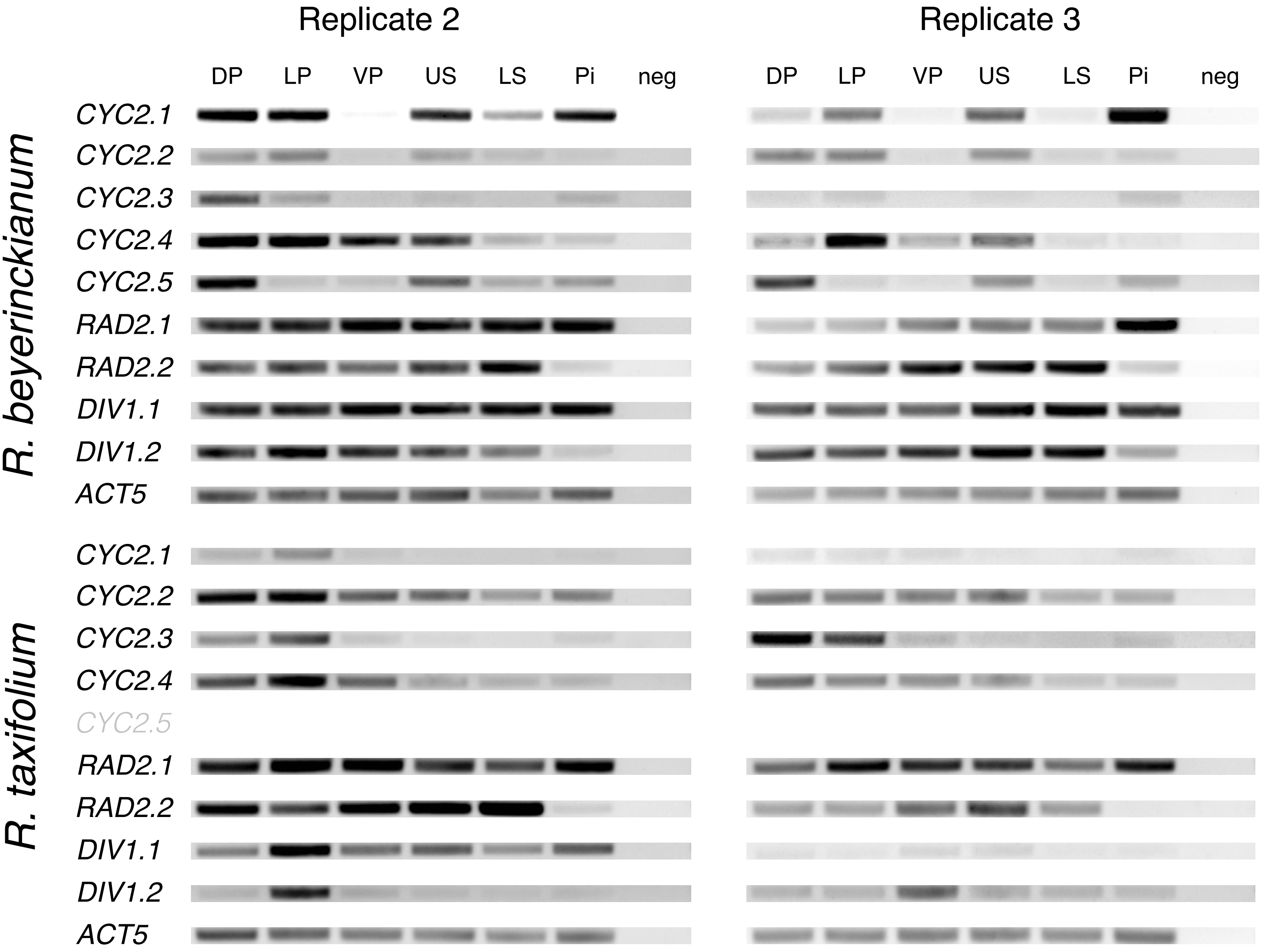

Supplementary Figure S6. **Gene expression of *CYCLOIDEA* (*CYC*), *RADIALIS* (*RAD*), and *DIVARICATA* (*DIV*) orthologs across floral organs in two *Rhododendron* species with different floral symmetries.** Floral organs sampled from late floral buds are dorsal petals (DP), lateral petals (LP), ventral petals (VP), upper stamens (US), lower stamens (LS), and pistils (Pi). Gene expression shown by RT-PCR for two additional replicates. Negative (neg) controls also shown. *ACTIN5* (*ACT5*) used as a reference gene. *R. beyerinckianum* is bilaterally symmetric; *R. taxifolium* is radially symmetric.
