# Supplementary material for: Gene Duplication and Differential Expression of Flower Symmetry Genes in Rhododendron (Ericaceae)
Source: Plants (Basel). 2021 Sep 23;10(10):1994. doi: 10.3390/plants10101994 (PMC8541606; doi:10.3390/plants10101994)
Supplement: Supplementary file 1 [file plants-10-01994-s001.zip › Supplementary_tables.pdf]

**Supplementary Table S1.** ***Rhododendron*** **samples and outgroups collected for this study.**

| **Taxon** | **Collection locality** | **Herbarium voucher (Herbarium)** |
|---|---|---|
| *R. beyerinckianum* Koord. | Rhododendron Species Botanical Garden, Federal Way, WA, USA, accession 1985/047 | Soza et al. 1926 & 1933 (WTU) |
| *R. camtschaticum* Pall. | Rhododendron Species Botanical Garden, Federal Way, WA, USA, accession 1977/080 | Soza & Ramage 1934 (WTU) |
| *R. edgeworthii* Hook. f. | Rhododendron Species Botanical Garden, Federal Way, WA, USA, accession 2001/229 | Soza 1958 (WTU) |
| *R. meliphagidum* J.J.Sm. | Royal Botanic Garden Edinburgh, UK, accession 19880517 | Conlon C114 (E) |
| *R. taxifolium* Merr. | Rhododendron Species Botanical Garden, Federal Way, WA, USA, accession 2013/241 | Soza & Ramage 1954 (WTU) |
| *Empetrum nigrum* L. | University of Washington Medicinal Herb Garden, Seattle, WA, USA | Soza 1957 (WTU) |
| *Erica scoparia* L. | University of Washington Botanic Gardens, Washington Park Arboretum, Seattle, WA, USA, accession 303-81 | N/A |
| *Kalmia latifolia* L. | University of Washington Botanic Gardens, Washington Park Arboretum, Seattle, WA, USA, accession 1054-40 | Herbarium Committee 1935 (WTUH) |

**Supplementary Table S2. Sources of DNA sequences used in this study.**

GenBank accessions numbers indicated below unless otherwise noted by footnotes.

| **Taxon** | **CYC1** | **CYC2** | **CYC3** | **RAD1/3**[*] | **RAD2** | **DIV1** | **DIV2** | **DIV3** | ***ACT5*** |
|---|---|---|---|---|---|---|---|---|---|
| *R. beyerinckianum* | N/A | CYC2.1: MZ208772; CYC2.2: MZ208773; CYC2.3: MZ208774; CYC2.4: MZ208775; CYC2.5: MZ208776 | N/A | N/A | RAD2.1: MZ209225; RAD2.2: MZ209233 | DIV1.1: MZ209241; DIV1.2: MZ209249 | N/A | N/A | |
| *R. camtschaticum* | N/A | CYC2.1: MZ208777; CYC2.2: MZ208778; CYC2.3: MZ208779 | N/A | N/A | RAD2.1: MZ209227; RAD2.2: MZ209235 | DIV1.1: MZ209243; DIV1.2: MZ209251 | N/A | N/A | N/A |
| *R. delavayi* Franch.[1] | CYC1.1: scaffold387 | CYC2.1: scaffold376 | scaffold2478 | scaffold5710 | RAD2.1: scaffold9 | DIV1.1: scaffold119 | scaffold2143 | scaffold287210 | scaffold4208 |

| | | | | | | | | | |
|---|---|---|---|---|---|---|---|---|---|
| | 7 (DUH023932.1); CYC1.2: scaffold1078 (DUH000978.1); CYC1.3: scaffold6605 (DUH029120.1) | 1 (DUH023544.1); CYC2.2: scaffold3761 (DUH023545.1); CYC2.3: scaffold3761 (DUH023546.1); CYC2.4: scaffold3761 (DUH023547.1); CYC2.5: scaffold22592 | (DUH008840.1) | (DUH027555.2, DUH027548.1), scaffold6823 (DUH029449.1), scaffold287816 (DUH020051.1), scaffold6003 (DUH027870.1), scaffold5710 | (DUH032037.1); RAD2.2: scaffold314 (DUH022018.1) | 9 (DUH001881.1); DIV1.2: scaffold2184 (DUH007695.1) | (DUH007400) | (DUH013168.2), scaffold287745 (DUH018833.1) | (DUH025269.1) |
| *R. edgeworthii* | N/A | CYC2.1: | N/A | N/A | RAD2.1: | DIV1.1: | N/A | N/A | N/A |

| | | | | | | | | | |
|---|---|---|---|---|---|---|---|---|---|
| | | MZ208780; CYC2.2: MZ208781; CYC2.3: MZ208782; CYC2.4: MZ208783; CYC2.5: MZ208784; CYC2.6: MZ208785 | | | MZ209228; RAD2.2: MZ209236 | MZ209244; DIV1.2: MZ209252 | | | |
| *R. meliphagidum* | N/A | CYC2.1: MZ208786; CYC2.2: MZ208787; CYC2.3: MZ208788; CYC2.4: MZ208789 | N/A | N/A | RAD2.1: MZ209229; RAD2.2: MZ209237 | DIV1.1: MZ209245; DIV1.2: MZ209253 | N/A | N/A | N/A |
| *R. simsii* Planch.[2] | CYC1.1: CM024964.1 | CYC2.1: CM024962.1 | CM024961.1 (Rhsim09G | CM024958.1 (Rhsim06G | RAD2.1: CM024963.1 (Rhsim11G017 | DIV1.1: CM024953.1 | CM024957.1(Rhsim05G02 | CM024961.1 (Rhsim09G | N/A |

| | | | | | | | | | |
|---|---|---|---|---|---|---|---|---|---|
| | (Rhsim12G0144800); CYC1.2: CM024960.1 (Rhsim08G0137600) | (Rhsim10G0063600); CYC2.2: CM024962.1; CYC2.3: CM024962.1 (Rhsim10G0063800); CYC2.4: CM024962.1 (Rhsim10G0063900); CYC2.5: CM024962.1 (Rhsim10G0064000) | 0112900) | 0128900), CM024958.1 (Rhsim06G0032200), CM024959.1 (Rhsim07G0240600), CM024959.1 (Rhsim07G0239900) | 7900); RAD2.2: CM024957.1 (Rhsim05G0073900 | (Rhsim01G0235000); DIV1.2 CM024965.1 (Rhsim13G0072600 | 06600) | 0172100), CM024955.1 (Rhsim03G0139800) | |
| *R. taxifolium* | N/A | CYC2.1: MZ208790; | N/A | N/A | RAD2.1: MZ209226; | DIV1.1: MZ209242; | N/A | N/A | |

| | | | | | | | | | |
|---|---|---|---|---|---|---|---|---|---|
| | | CYC2.2: MZ208791; CYC2.3: MZ208792; CYC2.4: MZ208793 | | | RAD2.2: MZ209234 | DIV1.2: MZ209250 | | | |
| *R. williamsianum* Rehder & E.H. Wilson[3] | CYC1.2: LG01_ordered_scaffold_52 (C3L33_00958-RA); CYC1.1: unclustered_scaffold_797 (C3L33_23136-RA) | CYC2.1: LG11_ordered_scaffold_106 (C3L33_18671-RA); CYC2.2: LG11_ordered_scaffold_106 (C3L33_18672-RA); CYC2.3: LG11_ordered_scaffold_107 | LG10_ordered_scaffold_29 (C3L33_17041-RA) | LG06_ordered_scaffold_66, LG06_ordered_scaffold_126 (C3L33_11058-RA), LG05_ordered_scaffold_118 (C3L33_09508-RA), LG05_ordered_scaffold_118 | RAD2.1: LG13_ordered_scaffold_4 (C3L33_20823-RA); RAD2.2: LG12_ordered_scaffold_33 (C3L33_19524-RA) | DIV1.1: LG03_ordered scaffol_14 (C3L33_04512-RA); DIV1.2: LG04_ordered_scaffold_90 (C3L33_07065-RA) | LG12_ordered_scaffold_110 (C3L33_20406-RA) | LG10_ordered_scaffold_14 (C3L33_16627-RA), LG02_ordered_scaffold_91 (C3L33_02859-RA) | LG02_ordered_scaffold_83 (C3L33_02818-RA) |

| | | | | | | | | | |
|---|---|---|---|---|---|---|---|---|---|
| | | (C3L33_18673-RA) | | (C3L33_09514-RA) | | | | | |
| *Actinidia chinensis* Planch.[4] | N/A | Contig01177 (Actinidia24782), Lachesis_group4 (Actinidia36307), Lachesis_group11 (Actinidia18122) | N/A | N/A | Lachesis_group7, Lachesis_group12 (Actinidia00529) | Lachesis_group15 (Actinidia34447), Lachesis_group9 (Actinidia23898), Lachesis_group24 (Actinidia38831), Lachesis_group20 (Actinidia25144) | N/A | N/A | N/A |
| *Antirrhinum majus* L.[5] | *TCP5*: Chr5 (Am05g30260) | *CYC*: Y16313; *DICH*: | *TCP1*: Chr3 (Am03g341 | *RADL1*: AJ791699.1; | *RAD*: AY954971.1 | *DIV*: AY077453.1; | N/A | N/A | N/A |

| | | | | | | | | | |
|---|---|---|---|---|---|---|---|---|---|
| | | AF199465 | 20) | *RADL2*: Am05g02290 *RADL3*: DQ375227.1; *RADL4*: Am07g10810 *RADL5*: AJ793240.1 | | *DIVL1*: AY077454.1 | | | |
| *Aquilegia caerulea* E.James[6] | *CYC1:* Aqcoe3G395500.1; *CYC2*: Aqcoe3G048600.1 | N/A | N/A | N/A | N/A | N/A | N/A | N/A | N/A |
| *Arabidopsis thaliana* (L.) Heynh. | *TCP18*: NM_112741 | *TCP1*: NM_001160982 | *TCP12*: NM_105554 | *RL3*: NM_119820; *RL4*: NM_00108 | *RL1*: NM_120086; *RL2* (NM_127736) | N/A | N/A | N/A | N/A |

| | | | | | | | | | |
|---|---|---|---|---|---|---|---|---|---|
| | | | | 4443; *RL5*: NM_101808; *RL6*: NM_0010844356 | | | | | |
| *Empetrum nigrum* | N/A | MZ208769 | N/A | N/A | RAD2.1: MZ209230; RAD2.2: MZ209238 | DIV1.1: MZ209246; DIV1.2: MZ209254 | N/A | N/A | N/A |
| *Erica scoparia* | N/A | MZ208770 | N/A | N/A | RAD2.1: MZ209231; RAD2.2: MZ209239 | DIV1.1: MZ209247; DIV1.2: MZ209255 | N/A | N/A | N/A |
| *Kalmia latifolia* | N/A | MZ208771 | N/A | N/A | RAD2.1: MZ209232; RAD2.2: MZ209240 | DIV1.1: MZ209248; DIV1.2: MZ209256 | N/A | N/A | N/A |
| *Magnolia grandiflora* L.[7] | N/A | N/A | N/A | *RAD1*: WBOD-2086252; | N/A | *DIV1*: WBOD-2022953; *DIV2*: | N/A | N/A | N/A |

| | | | | | | | | | |
|---|---|---|---|---|---|---|---|---|---|
| | | | | *RAD2*: WBOD-2019537; *RAD3*: WBOD-2006094 | | WBOD-2024364 | | | |
| *Solanum lycopersicum* L.[6] | *CYC1A*: Solyc03g119770.2.1; *CYC1B*: Solyc06g069240.1.1 | *CYC2A*: Solyc02g089830.1.1; *CYC2B*: Solyc03g045030.1.1 | *CYC3A*: Solyc05g009900.1.1; *CYC3B*: Solyc04g006980.1.1 | *RADL5*: Solyc10g080920.1.1; *RADL3*: Solyc04g080500.2.1; *RADL4*: Solyc12g089190.1.1; *RADL1*: Solyc01g109690.1.1 | *RAD*: Solyc01g109670.2.1; *RADL2*: Solyc10g052470.1.1 | *DIV*: Solyc06g076770.2.1 | *DIVL*: Solyc09g014250.2.1; *DIVL2*: Solyc10g076820.1.1 | *DIVL4*: Solyc03g096350.2.1; *DIVL5*: Solyc05g052610.2.1 | N/A |
| *Vaccinium corymbosum* L.[8] | N/A | VaccDscaff7-snap-gene-330.28, | N/A | N/A | VaccDscaff14-gene-372.36, VaccDscaff2-s | VaccDscaff19-augustus-gene-360.1 | N/A | N/A | N/A |

| | | | | | | | | | |
|---|---|---|---|---|---|---|---|---|---|
| | | VaccDscaff16-snap-gene-95.39, VaccDscaff31-snap-gene-5.36 | | | nap-gene-426.30, VaccDscaff13-snap-gene-273.47, VaccDscaff30-snap-gene-127.34, VaccDscaff32-processed-gene-84.20, VaccDscaff42-processed-gene-185.4 | 9, VaccDscaff15-augustus-gene-332.31, VaccDscaff15-augustus-gene-315.33, VaccDscaff24-augustus-gene-73.38 | | | |
| *Vitis vinifera* L.[6] | GSVIVT01008234001 | GSVIVT01036449001 | GSVIVT01011962001 | *RADL4*: GSVIVT01009723001; *RADL2*: | *RAD*: GSVIVT01031975001 | *DIV*: GSVIVG01024794001 | *DIVL2*: GSVIVG01034001001 | *DIVL3*: GSVIVG01016370001 | N/A |

| | | | | | | | | | |
|---|---|---|---|---|---|---|---|---|---|
| | | | | GSVIVT01 018944001; *RADL1*: GSVIVT01 031982001; *RADL3*: GSVIVT01 009716001 | | | | | |

[*]RAD1 and RAD 3 are not supported as distinct groups.

[1]All *R. delavayi* sequences obtained from Zhang et al. 2017 at http://gigadb.org/dataset/100331. Genomic scaffold and gene ID, if predicted, given.

[2]All *R. simsii* sequences obtained from Yang et al. 2020 at http://bioinfor.kib.ac.cn/RPGD/index.html. Genomic scaffold and gene ID, if predicted, given.

[3]All *R. williamsianum* sequences obtained from Soza et al. 2019 at https://genomevolution.org/coge/GenomeInfo.pl?gid=59084. Genomic scaffold and gene ID, if predicted, given.

[4]All *A. chinensis* sequences obtained from Wu et al. 2019 at http://kiwifruitgenome.org/organism/5. Genomic scaffold and gene ID, if predicted, given.

[5]*A. majus* sequences obtained from GenBank or Li et al. 2019 at http://bioinfo.sibs.ac.cn/Am. Genomic scaffold and gene ID, if predicted, given.

[6]All sequences obtained from Phytozome at https://phytozome.jgi.doe.gov/pz/portal.html.

[7]All *M. grandiflora* sequences obtained from 1,000 Plants (Carpenter et al. 2019, Leebens-Mack et al. 2019) at China National Gene Bank, https://db.cngb.org.

[8]All *V. corymbosum* sequences obtained from Colle et al. 2019 at https://www.vaccinium.org. Genomic scaffold and gene ID, if predicted, given.

**Supplementary Table S3. Primer sequences and thermal cycling conditions used in this study.**

| Primer | Sequence | Annealing Temperature (C) | Extension Time (s) | Purpose |
|---|---|---|---|---|
| **CYC2** | | | | |
| RhCYC2A_StartF2 | 5'-ATGWTCTCTTCAACCAMYAAYGS-3' | 62 | 60 | Ingroup PCR |
| RhCYC2A_StopR | 5'-GTTACCAYTGGCTGWAGATGTTGC-3' | 62 | 60 | Ingroup PCR |
| RhCYC2_TCPF | 5'-GCACAAGCACAGYAAGATYT-3' | 52 | 30 | Outgroup PCR |
| RhCYC2_RR | 5'-CTCTCTHGCYYTTRCCCTAGA-3' | 52 | 30 | Outgroup PCR |
| IDT ReadyMade™ Anchored Oligo dT (20) | 5'-TTTTTTTTTTTTTTTTTTTV-3' | 45/55 | 180 | 3' RACE |
| Rbey_CYC2_3RACE_F1 | 5'-TTCTTCSTCCAGTACCACTC-3' | 45/55 | 180 | 3' RACE |
| Rbey_CYC2_3RACE_F2 | 5'-YAWGCTAGGGTTCGAGAAAG-3' | 45/55 | 180 | 3' RACE |
| *RhCYC2.1* | | | | |
| Rbey_CYC2_3RACE_intF | 5'-TCTACAGCCAATGGTGACAT-3' | 50 | 240 | 3' RACE sequencing |
| Rbey_CYC2.1B_RT_F | 5'-CGCTGGCAAGTTCTTCGATCTT-3' | 68 | 30 | RT-PCR |
| Rbey_CYC2.1B_RT_F2 | 5'-GGTGAACCCAATGGTGAAA-3' | 60 | 90 | 3' UTR PCR |
| Rbey_CYC2.1_RT_R | 5'-GGCCGGCTCAAGAGTTTT-3' | 68, 60 | 30, 90 | RT-PCR, 3' UTR PCR |
| Rbey_CYC2.1_intF | 5'-GAGTGACGTGGATCAAGTAT-3' | 50 | 240 | 3' UTR sequencing |
| Rbey_CYC2.1_intF2 | 5'-GCTATTGAGCCAAAGGCCTC-3' | 50 | 240 | 3' UTR sequencing |
| Rbey_CYC2.1_intR | 5'-TCCTAGGGAGAGAGAGAGAG-3' | 50 | 240 | 3' UTR sequencing |
| Rbey_CYC2.1_intR2 | 5'-TCACAACCGTAAACACGATC-3' | 50 | 240 | 3' UTR sequencing |

| | | | | |
|---|---|---|---|---|
| Rtax_CYC2.1_RT_F2 | 5'-AAGCCGGTGAACCCAATGGT-3' | 64 | 30 | RT-PCR |
| Rtax_CYC2.1_RT_R2 | 5'-GACTTTACACCACCACTYGAG-3' | 64 | 30 | RT-PCR |
| *RhCYC2.2* | | | | |
| Rbey_CYC2.2_F | 5'-ATGGTGTAAAGGGGAGTACTCT-3' | 64 | 90 | 3' UTR PCR |
| Rbey_CYC2.2_RT_F2 | 5'-CAACTTGGAGGATGGAGCAT-3' | 65 | 30 | RT-PCR |
| Rbey_CYC2.2_RT_R2 | 5'-GCCTACCCGTAACCCAAAA-3' | 65, 64 | 30, 90 | RT-PCR, 3' UTR PCR |
| Rtax_CYC2.2_RT_F | 5'-CTTTTCGGCATGGTGGTTTC-3' | 64 | 30 | RT-PCR |
| Rtax_CYC2.2_RT_R | 5'-GCTGATACCACTTGATCACTTCC-3' | 64 | 30 | RT-PCR |
| *RhCYC2.3* | | | | |
| Rbey_CYC2.3_RT_F | 5'-CCTGACCGACACCCTTTT-3' | 65 | 30 | RT-PCR |
| Rbey_CYC2.3_RT_R | 5'-CAGCAGCCCTATCTTTAAGT-3' | 65 | 30 | RT-PCR |
| Rtax_CYC2.3_RT_F | 5'-GTCGATCGAAACGGCTCGCAAGTTC-3' | 66 | 30 | RT-PCR |
| Rtax_CYC2.3_RT_R | 5'-ATGCAGCAGCCCTATCTTTAAGTAA-3' | 66 | 30 | RT-PCR |
| *RhCYC2.4* | | | | |
| Rbey_CYC2.4a_RT_F | 5'-TCCAAGTTGAAGCGATCTCC-3' | 67 | 90 | 3' UTR PCR |
| Rbey_CYC2.4c_RT_F | 5'-AGGTTCTGTTGTGGGCATTA-3' | 67 | 30 | RT-PCR |
| Rbey_CYC2.4c_RT_R | 5'-TAGCCAGAGAGCCAAACAAA-3' | 67 | 30, 90 | RT-PCR, 3' UTR PCR |
| Rtax_CYC2.4_3utrF | 5'-GACACAGAAAGATAGGACTGCT-3' | 58 | 90 | 3' UTR PCR |
| Rtax_CYC2.4_3utrR | 5'-CAAGAGGTAATATTGCGGCAG-3' | 58 | 90 | 3' UTR PCR |
| Rtax_CYC2.4_RT_F | 5'-AAGAAGGCCGCGAAGAAG-3' | 66 | 30 | RT-PCR |
| Rtax_CYC2.4_RT_R | 5'-AGCAGTCCTATCTTTCTGTGTC-3' | 66 | 30 | RT-PCR |

| | | | | |
|---|---|---|---|---|
| *RhCYC2.5* | | | | |
| Rbey_CYC2.5_3RACE_F2 | 5’-TCAGGGACCAATATTGAAGGT-3’ | 45/55, 66 | 180, 90 | 3’ RACE, 3’ UTR PCR |
| Rbey_CYC2.5c_3RACE_R | 5’-ATCATGGTACATCGGCACTA-3’ | 66 | 90 | 3’ UTR PCR |
| Rbey_CYC2.5_intF | 5’-ATACGAAAATGGCAGTGTCT-3’ | 50 | 240 | 3’ UTR sequencing |
| Rbey_CYC2.5_intR | 5’-CCAAGACAACAAAACACCTC-3’ | 50 | 240 | 3’ UTR sequencing |
| Rbey_CYC2.5_RT_F | 5'-CGAGAAACCAAGAAGCCAG-3' | 65 | 30 | RT-PCR |
| Rbey_CYC2.5_RT_R | 5'-ACCTTCAATATTGGTCCCTGA-3' | 65 | 30 | RT-PCR |
| **RAD2** | | | | |
| *RhRAD2.1* | | | | |
| RhRADcopy1_F1 | 5’-ACGTCCTCCCAAGGCTCTG-3’ | 67 | 35 | PCR |
| RhRADcopy1_R1 | 5’-GGTCTTGTAGTCAGGAAAGGG-3’ | 67 | 35 | PCR |
| RhRADcp1_fwd_rtPCR | 5'-ACCATCTACTCTACTTTCCG-3' | 56 (Rtax) or 61 (Rbey) | 30 | RT-PCR |
| RhRADcp1_rev2_rtPCR | 5'-CCTTTGCTTCTCTTCACTAG-3' | 56 (Rtax) or 61 (Rbey) | 30 | RT-PCR |
| *RhRAD2.2* | | | | |
| RhRADcopy2_F1 | 5’-ATGGCATCAATGTCTTCCG-3’ | 67 | 35 | PCR |
| RhRADcopy2_R1 | 5’-AGTTCTTGTAGTTTGGATAGG-3’ | 67 | 35 | PCR |
| RhRADcp2_fwd1_rtPCR | 5'-CCTTCTCACTCTACCGAAACAT-3' | 56 (Rtax) or 61 (Rbey) | 30 | RT-PCR |
| RhRADcp2_rev1_rtPCR | 5'-CCTTGGTTCATCACTTCATCT-3' | 56 (Rtax) or 61 (Rbey) | 30 | RT-PCR |
| **DIV1** | | | | |
| *RhDIV1.1* | | | | |
| RhDIV_LG03_F1 | 5’-ATGAGGTGGGGAATGGAGATTC-3' | 64 | 35 | PCR |
| RhDIV_LG03_R1 | 5’-CTGACTTGTGCTCTTCTTCAG-3' | 64 | 35 | PCR |

| | | | | |
|---|---|---|---|---|
| RhDIVlg03_fwd2 | 5'-GCTCCTCTGCCTTCTCTTG-3' | 61 (Rtax) or 63 (Rbey) | 30 | RT-PCR |
| RhDIVlg03_rev1 | 5'-GGAAGAAGCACTATTATACCACG-3' | 61 (Rtax) or 63 (Rbey) | 30 | RT-PCR |
| *RhDIV1.2* | | | | |
| RhDIV_LG04_F1 | ATGGAAATTCTGTCGCCTGCATC | 62 | 35 | PCR |
| RhDIV_LG04_R1 | CCAACTTACTTATGCTCTTCTTCTG | 62 | 35 | PCR |
| RhDIVlg04_fwd1 | 5'-GCTGTGAGGTGGGTTTTCC-3' | 63 (Rbey), 66 (Rtax) | 30 | RT-PCR |
| RhDIVlg04_rev1 | 5'-CGGGAACCACTGTACCAAG-3' | 63 (Rbey), 66 (Rtax) | 30 | RT-PCR |
| ***ACT5*** | | | | |
| DN129728_c6_g2_i3_Fwd_primer3 | 5’-CGGGGTTGGATGAAATGGCT-3’ | 63 (Rtax) or 65 (Rbey) | 30 | RT-PCR |
| DN129728_c6_g2_i3_Rev_primer3 | 5’-GCCAATCCTCACCCTTCTCA-3’ | 63 (Rtax) or 65 (Rbey) | 30 | RT-PCR |
| ***EF1A*** | | | | |
| RhEF1a_Yi_FWD | 5’-TGTCATCGATGCTCCTGGAC-3' | 62 | 30 | RT-PCR |
| RhEF1a_REV | 5’-AGGGCCTCTTGGGTTCACTAA-3' | 62 | 30 | RT-PCR |
| **M13** | | | | |
| M13*F | 5’-GTAAAACGACGGCCAGTGAAT-3’ | 55 | 30 or 60 | Clone screening |
| M13*R | 5’-CAGGAAACAGCTATGACCATG-3’ | 55 | 30 or 60 | Clone screening |
